# Management strategies of dental anxiety and uncooperative behaviors in children with Autism spectrum disorder

**DOI:** 10.1186/s12887-023-04439-7

**Published:** 2023-12-04

**Authors:** Shi-Jun Tang, Hong-Lin Wei, Cai-Yu Li, Ming-Na Huang

**Affiliations:** Department of Pediatric Dentistry, Stomatological Hospital of Guizhou Medical University, Guiyang, China

**Keywords:** Dental anxiety, Autism spectrum disorder, Pediatrics

## Abstract

**Background:**

Children with Autism spectrum disorder (ASD) was frequently experienced dental anxiety and uncooperative behaviors during dental treatment. Oral health care was necessary because of the poor oral hygiene and prevalent dental diseases in this population.

**Aim:**

In this systematic review, we evaluated the effectiveness and feasibility for pediatric dentist to manage the dental anxiety in children with ASD.

**Design:**

PubMed, Embase, and Cochrane Library were systematically performed on the literature search. The date of eligible publications was from inception to January 2023. After that, the quality of eligible studies was assessed by the Newcastle Ottawa Scale (NOS). Review findings were summarized using the PRISMA Statement for reporting.

**Results:**

A total of six studies were systematically evaluated according to the inclusion and exclusion criteria. Five studies were conducted to evaluate ASD Children's anxiety and uncooperative performance in the progressive oral examination, oral disease prophylaxis and fluoride application. The other one study evaluated the success rate of treatment in decayed permanent tooth treatment. In the included studies, four studies indicated that it was extremely necessary to reduce dental anxiety of ASD children to increase the cooperation in sensory-adapted dental environment (SADE).

**Conclusion:**

It is not always effective and feasible for pediatric dentist to manage the dental anxiety in children with autism during routine oral examination. Meanwhile, it is necessary for ASD children to conduct preoperative psychological assessment, to investigate parents’ expectations and cooperation, and to determine whether to start corresponding dental treatment.

**Supplementary Information:**

The online version contains supplementary material available at 10.1186/s12887-023-04439-7.

## Introduction

Autism spectrum disorder (ASD), characterized by impaired social interaction, communication deficits, limited interest, and the presence of repetitive and stereotyped behaviors, is a complex neurodevelopmental condition, especially in children [1]. Compared to typically developing(TD) children, dental anxiety and fear were often existed in a group of children with ASD, as manifested via difficult behaviors and uncooperative reactions during dental treatment [2–4]. These uncooperative behaviors and uncontrolled body movements (including hyperactivity, impulsivity, anger, self- stimulatory, selfinjurious and disruptive behaviors) make dental treatment more complex [5, 6].

Dental care and oral health, for children with such disorders, were areas that required special procedures and adaptations because of the poor oral hygiene and severe dental diseases in this population [7]. Marshall et al. [8] found that the oral hygiene status of children with ASD was associated with risk indicators that had an impact on new dental caries. Dental treatment was full of potentially frightening stimuli due to the peculiar environment, unfamiliar pronunciations and strongly light exposure [1]. It had been proved that autistic children commonly exhibited uncooperative behaviors such as crying or physical aggression during dentist visits. One of the reasons which might lead to worse oral health was the dental anxiety about dental examination and oral hygiene status in ASD population, which impeded their oral care [9–11]. The focus of oral behavior management in pediatric dentistry was communication and demonstration of dental treatment procedures [12], children with ASD needed to formulate the better adapted and specific strategies to improve the success rate of dental care [13]. The use of visual pedagogy was an effective practice to improve cooperative behavior in children with ASD [14], and these could effectively reduce dental anxiety through video peer-to-peer modeling or difference types of electronic screen media temptation and intervention [15]. Cermak et al. [16] analyzed the pain, behavior and sensory discomfort both in a regular dental environment (RDE) and in a sensory-adapted dental environment (SADE) between ASD and TD children in period of dental treatment. Same with the result of another author, the research showed that a SADE might be effective in reducing anxiety and inducing relaxation [17].

According to the existing studies, due to the widespread development of general anesthesia for children's dental treatment, there was still controversy regarding whether it was necessary to induce to relieve dental anxiety and uncooperative behavior of autistic children, and what kind of behavior management was used for dental care and oral health. Therefore, we summarized and assessed the feasibility and effectiveness of this systematic review in managing dental anxiety and uncooperative behavior in autistic children who underwent oral health care and dental treatment.

## Methods

The current studies were conducted on the basis of the Preferred Reporting Items for Systematic Reviews and Meta-Analyses (PRISMA) statement [18]. The registration ID on PROSPERO is CRD42023473698, and the link of the PROSPERO is https://www.crd.york.ac.uk/ prospero/display_ record.php?ID=CRD42023473698.

### The project of research

The main question of the current research was carried out to determine in this systematic review through which the literature was investigated: How to alleviate dental phobia of autistic children, how to evaluate their degree of cooperation and how to take effective oral treatment?

### The PICO strategy

Answering the above-mentioned research questions through PICOs method which the clinical questions can be correctly formulated and derives from the Evidence Based Medicine (EBM) approach, was used to write systematic reviews. In this strategy, the study of following aspects should be considered:

- P: Population of interest (autistic children between 3 and 19 years old)
- I: Interventions (management techniques in the dental treatment and the cooperation rate of children)
- C: Control (TD children and ASD group in different dental environment)
- O: Outcome (the improvement of dental anxiety in the behavior scale)

### Literature search

The literature search strategy was performed in PubMed, Embase and the Cochrane Library (all from inception to January 2023). The search items were as follows: (“Autistic Disorder” OR “autism” OR “Asperger syndrome” OR “Autism Spectrum Disorder” OR “ASD”) AND (“dental fear” OR “dental anxiety” OR “dental anxieties” OR “dental phobia” OR “Dentophobia” OR “Odontophobia”). The title and abstract of all searched articles were independently screened by two authors. The search was restricted to English articles and human studies. Any duplicates or no relevance articles were rejected. Full texts of eligible articles were retrieved for further evaluation (Table 1).

**Table 1:** The search strategy of the review

Search strategy
Search Databases: PubMed, Embase, Cochrane Library
Date: As of Jan 3, 2023
Search term: ((((((dental fear) OR (dental anxiety)) OR (dental anxieties)) OR (dental phobia)) OR (Dentophobia)) OR (Odontophobia)) AND (((((Autistic Disorder) OR (autism)) OR (Asperger syndrome)) OR (Autism Spectrum Disorder)) OR (ASD))

### Inclusion and exclusion criteria

The research scope of this systematic review that met the following criteria were eligible for inclusion:

- 1) Articles in English;
- 2) Controlled trials, interventional study and studies with comparative groups in full text;
- 3) Children population suffering from ASD;
- 4) Interventions for behavior scales or cooperation rate;

The exclusion criteria were as follows:

- 1) Absence of English language;
- 2) Meta-analyses, reviews and case reports;
- 3) Children population with other diagnoses or ASD adults;
- 4) Duplicate studies.

### Data extraction

Data information was repeatedly confirmed and extracted by two authors, the following detailed analysis was listed: descriptive characteristics (authors information and study design), participants, comparative groups, assessment tools and dental procedures in each included study. The significance and effectiveness in the anxiety and behavior scores and the effect comparison before and after intervention were evaluated according to the management of intervention and measurement of result.

### Quality assessment

The quality assessment of the six included studies was conducted by the Newcastle-Ottawa Scale (NOS). As for the NOS score, high quality was scored greater than seven; otherwise, it was low quality [19]. The results were showed in Table 2.

**Table 2:** Results of quality assessment using the Newcastle–Ottawa Scale for cohort studies

Study	Selection				Comparability	Outcome			Scores
	Representa-tiveness of exposure	Selection of the non- exposure	Ascertainment of exposure	Demonstration that outcome was not present at start	Cohorts on the basis of the design or analysis	Assessment	Long follow-up for outcomes to occur	Adequacy of follow up	
Isong, 2014 [15]	★	★	★	★	★ ★	★	★	★	9
Stein, 2014 [7]	☆	★	★	★	★ ★	★	★	★	8
Cermak,2015 [16]	★	★	★	★	★ ★	★	★	★	9
G.Lefer,2018 [13]	★	★	★	★	★ ★	☆	★	★	8
YePark, 2022 [22]	★	★	★	★	★ ★	☆	★	★	8
Fallea, 2022 [29]	☆	★	★	★	★ ★	☆	★	★	7

Comparability has two scores, and the other items only have one score. The black stars symbol means yes, or the white stars symbol means no

## Result

### Study selection

**Fig. 1:**
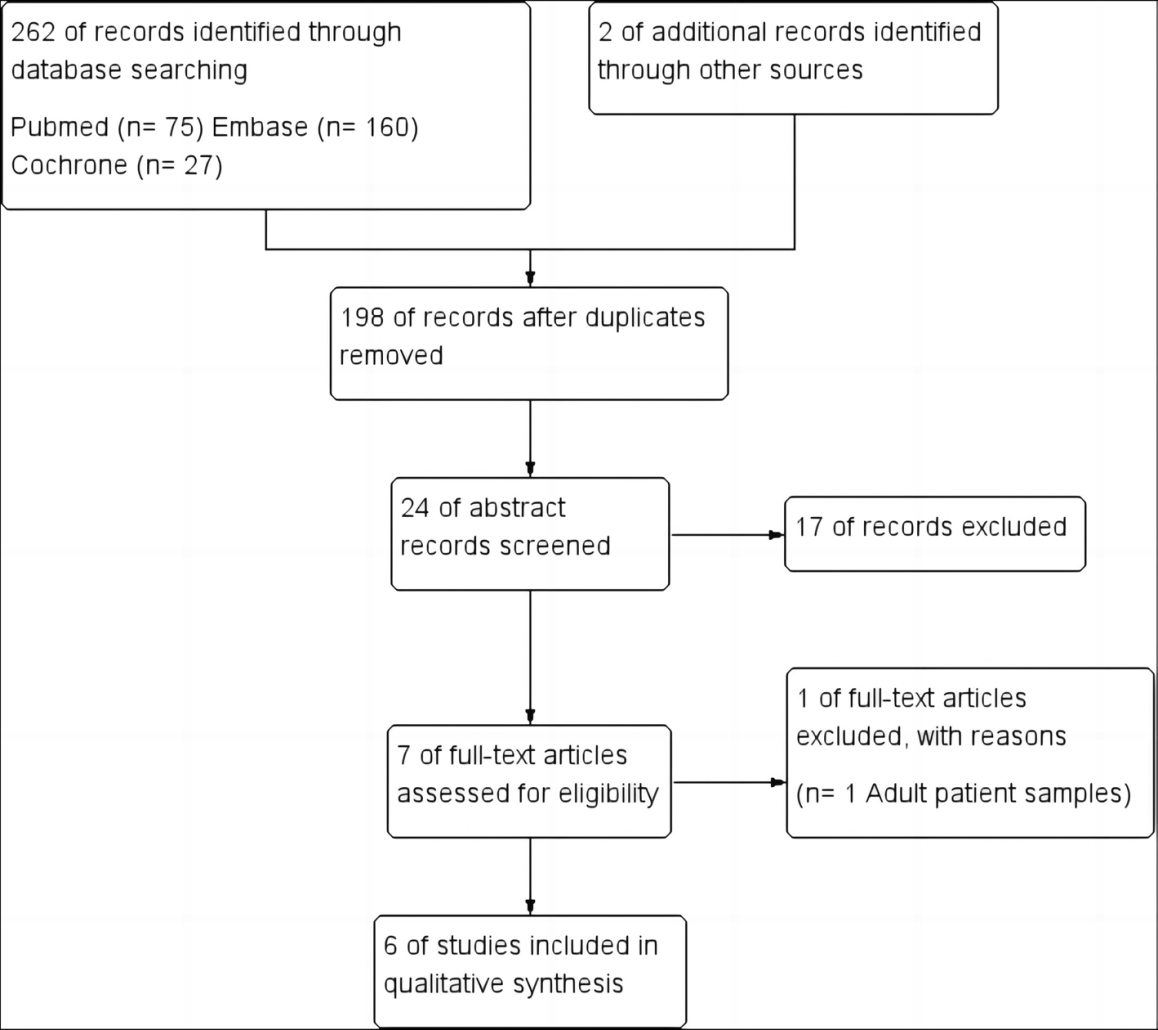
The flow diagram of studies identified in the systematic review

The electronic search resulted in 264 studies, of which 66 studies were duplicates and eliminated. The abstracts of 198 articles were screened, and the related studies were read by both researchers for potential inclusion. Seven full-texts were assessed for eligibility, and one study was excluded for the following reasons: adult samples with ASD. Finally six studies were included in this systematic literature review. According to the PRISMA statement, a detailed flowchart in Figure 1 was developed for the screening process of the study.

### Descriptive characteristics

All included studies were organized based on different intervention approach. Publication year of studies ranged from 2014 to 2022. The overall sample sizes in these studies varied from 44 to 80 and the total number of child participants were 346, of which 302 were autistic children. Each study had different and diverse tools that were used to assess the influence of intervention based on the performance of behavior scales or cooperation rate during dental assessment. Five studies were conducted to evaluate anxiety and cooperative behavior in children with ASD by the progressive oral examination, fluoride application and oral disease prophylaxis. The other one study evaluated the success rate of treatment in decayed permanent tooth treatment.

We divided their conclusions on reduction of dental anxiety in children with ASD by summarizing the main results of six studies. Four studies indicated that it was extremely necessary to reduce dental anxiety of ASD children to increase the cooperation in SADE. Besides, mean anxiety and behavioral scores significantly decreased according to the application of video modeling plus video goggles/DVD. However, one of the studies was concluded that there were no significant correlations between the Abeer Children Dental Anxiety Scale (ACDAS) and any hypothetical variables (Table 3).

**Table3:** Descriptive characteristics of the included studies and summary of results

First Author (Year of Publication)	Study Design	Study Participants	Participants’ Medical Status	Dependent variables	Dental Procedure	Results	NOS
Isong, 2014 [15]	RCT	80(7-17 years old)	Four groups with ASD Group A: control group; group B: only video peer modeling ; group C: only video goggles/DVD ; group D: B+C	Venham Anxiety and Behavior Scores	oral examination, radiographs, scaling (if needed), prophylaxis, application of fluoride	The average anxiety and behavioral scores of group C subjects decreased significantly by 0.9 points, while the average anxiety and behavioral scores of group D subjects decreased by 0.8 points in visit 1 and visit 2, respectively. There were no significant changes in the average anxiety and behavioral scores of subjects in Group A and Group B.	9
Stein, 2014 [7]	CCT	44(6-12 years old)	One group with ASD One group with TD	A & C scale, Children’s Dental Behavior Rating Scale(CDBRS)	oral examination, prophylaxis (cleaning), application of fluoride.	Children with ASD had more difficulties in sensory processing, general anxiety, and dental anxiety than children with TD Children with ASD demonstrated greater resistance and uncooperative behavior at the time of dental healthcare, compared to the TD group	8
Cermak,2015 [16]	RCT	44(6-12 years old)	One group with ASD in RED One group with ASD in SADE One group with TD in RED One group with TD in SADE	CDBRS scaled, A & C scale, Frankl scale, Pain intensity	Oral examination, prophylaxis(cleaning), fluoride application	The Anxiety and Cooperation scale Showed that SADE conditions could improve the relaxation and cooperation rate of the ASD group (46 % RDE to 59 % SADE) and most TD children exhibited positive dental cooperative behavior in both environmental conditions (91 and 95 % for RDE and SADE, respectively).	9
G.Lefer,2018 [13]	prospective	52(3-19 years old)	All Children with ASD(dental exam at the beginning baseline of the study (T0) and every two months thereafter (T1, T2, T3, and T4)	Scores of achievement (SAch), Scores of anxiety (SAnx)	Progressive dental exam: Sitting→Light shining→mouth opening→Placing a mirror into the mouth→Placing an explorer on the teeth→Placing both the mirror and explorer into the mouth	SAch scores increased significantly between T0 and T1, between T2 and T3, and between T0 and T4. For SAnx, the differences between T0 and T1 and between T0 and T4 are significant. The study sample made progress in all steps of achievement, and individuals became less anxious.	8
YePark, 2022 [22]	SM	76(7-13 years old)	All Children with ASD	Abeer Children Dental Anxiety Scale (ACDAS)	The most common feeling of dental anxiety in this sample, include: "pinching" of the gums, "tooth extraction", "wearing a gas mask is necessary," and "strange taste in the mouth."	there were no significant associations between the ACDAS and any hypothetical variables in all children with ASD.	8
Fallea, 2022 [29]	CCT	50(9-10 years old)	All Children with ASD(Oral therapy of each participant in RED and SADE)	Treatment success rate	Treat decayed permanent tooth	The treatment success rate of SADE group is higher than that of RDE group.	7

*Abbreviations*: *NOS* Newcastle Ottawa Scale, *RCT* Randomized clinical trial, *CCT* Controlled clinical trial, *SM* Split-mouth design

**Table 4:** Studies assessing CDBRS scaled and A & C scale in dependent variables

**Study**	**Dependent** **variables**	**TD** Mean ± SD		**ASD** Mean ± SD		**Effect size**	
Stein, 2014	CDBRS scale	34.69 (12.47)		47.31 (8.61)		1.1^∗^	
	A & C scale	0.45 ± 1.06		2.07 ± 1.59		1.3^∗^	
Cermak, 2015	CDBRS scale	**TD**		**ASD**		**Effect size**	
		RDE	SADE	RDE	SADE	TD	ASD
	A & C scale	Mean ± SD	Mean ± SD	Mean ± SD	Mean ± SD		
		34.7 ± 12.5	30.8 ± 14.6	47.3 ± 8.6	44.9 ± 11.8	0.29	0.23
		0.5 ± 1.1	0.4 ± 1.1	2.1 ± 1.6	1.8 ± 2.0	0.06	0.13

Conditions could improve the relaxation and cooperation rate of the ASD group (46 % RDE to 59 % SADE) and most TD children exhibited positive dental cooperative behavior in both environmental conditions (91 and 95 % for RDE and SADE, respectively) (Table 4)

We extracted Children’s Dental Behavior Rating Scale (CDBRS) and A & C scale outcome data from two studies that compared the dental anxiety and cooperation behavior outcomes of TD and ASD groups. The outcome reported by Leah I. Stein et al [14] indicated that compared to the TD group, children with ASD demonstrated greater resistance and uncooperative behavior at the time of dental healthcare, based on dentist-report on the Anxiety and Cooperation Scale and the Frankl Scale (both P’s < 0.001), as well as overt behaviors coded by the CDBRS (P = 0.001). The above results were consistent with those reported by Cermak et al [16]. In addition, there were data showing that SADE

## Discussion

In this research, we systematically reviewed six published scientific studies focusing on ASD Children's dental anxiety and cooperative behavior during oral examination and treatment. The overall sample size of these studies ranges from 44 to 80, with a total of 346 child participants, including 302 children with autism.

Research so far, this is the first systematic review to summarize the clinical practical significance of dental anxiety in ASD children by specific evaluation scales. In the five articles of this systematic review, the researchers reported that SADE could reduce dental anxiety of ASD children to increase the cooperation in the progressive oral examination, dental prophylaxis and fluoride application. In another study, the researchers recommended that ASD children had gradually better performed and less anxiety in dental exam at the beginning baseline of the study (T0) and every two months thereafter (T1, T2, T3, and T4).

Dental visit was a challenge thing for autistic children, parents sometimes delayed or refused to go to the dentist for autistic children because of dental fear and extremely uncooperative performance [20, 21]. According to a previous study that children with ASD might be particularly vulnerable to dental anxiety, about 70% of subjects experienced clinically significant dental anxiety, which could lead to uncooperative behavior at the dentist and refuse any dental examination. Thus, poor dental health was a common condition in this population [22]. Compared to TD children, ASD children showed more obvious behavioral abnormalities during dental care. This result was consistent with previous research reporting a series of uncooperative behaviors in dental treatment of autistic children [3, 23]. Meanwhile, this supported Dawson et al. [24] view that children with ASD would activate their sympathetic “fight or flight” nervous system in this time of stress, making them hate dental cleanings more than TD children. Furthermore, dental environment was also important, most studies showed that ASD children exhibited less physiological and behavioral manifestations of distress receiving routine oral cleanings at the SADE compared to the RDE.

In pediatric dentistry, conventional behavioral managements such as tell-show-do, voice control, nonverbal communication, and verbal positive reinforcement might be effective [12]. However, due to ASD children’s common communication barriers and lower expressive communication ability, new strategies might be applied to increase cooperative and controllable behavior in children with ASD compared to TD children. These managements such as using visual aids, behavioral training and modeling, pictures displaying, stories reading and desensitization might be helpful for children’s dental visit, and these technologies have been successfully applied to children with ASD in pediatric dentistry and other fields [15, 25–28]. In this systematic review, four studies [13, 15, 16, 29] were assessed to investigate the dental anxiety of ASD children with specific management strategies during dental care. In our included studies, G. Lefer et al. [13] reported that when performing this activity in progressive dental management, dental examinations were more effective and less anxiety was observed. The other three studies indicated that children reported significant improvements in pain intensity and sensory discomfort measurements in the dental environment in both the ASD and TD groups during SADE, also the treatment success rate of SADE group was higher than that of RDE group. Furthermore, the rest two studies [7, 22] used different scales to estimate the dental anxiety and uncooperative behavior. Stein et al. [7] reported that children with ASD demonstrated greater resistance and uncooperative behavior at the time of dental healthcare, compared to the TD group, based on dentist-report on the Anxiety and Cooperation Scale and the Frankl Scale, as well as by the CDBRS. According to parents' reports, children with autism spectrum disorders had more difficulties in sensory processing, general anxiety, and dental anxiety than children with TD in this study. However, Ye Park et al. [22], in their analysis, demonstrated that there were no significant associations between the ACDAS and any hypothetical variables in all children with ASD.

In this systematic review, although the dental anxiety and uncooperative behavior in autistic children might influence the decision and treatment methods of pediatric dentist to complete a full dental care and oral health, there was significant difference in the measures of sensory discomfort and pain intensity between the SADE and RDE groups. Therefore, it is necessary for the pediatric dentist to take appropriate management strategies to intervene in dental examination and treatment for children with ASD unless encountered great resistance from parents and children during the dental visit.

There are some limitations in the current review. First, the six studies included in this systematic review were restricted to English language searching, which would cause some important information to be lacking in other language. Second, these six studies lacked flexible data to implement a meta-analysis to support this research. Third, interventions and measurement scales in each study were inconsistent. Finally, the sample size was relatively small. Therefore, wider regional researches from more countries and randomized controlled trials verifying the relationship between dental anxiety and abnormal behavior in ASD children were required to confirm our results.

## Conclusion

It is not always effective and feasible for pediatric dentist to manage in dental care due to the stress of dental anxiety in children with ASD. Also, it is necessary for ASD children to conduct preoperative psychological assessment, to investigate parents’ expectations and cooperation, and determine whether to start corresponding dental treatment.

### Supplementary Information

**Additional file 1.** Addressing dental fear in children with autism spectrum disorders: a randomized controlled pilot study using electronic screen media

**Additional file 2.** Physiological and behavioral stress and anxiety in children with autism spectrum disorders during routine oral care

**Additional file 3.** Sensory adapted dental environments to enhance oral care for children with autism spectrum disorders: a randomized controlled pilot study

**Additional file 4.** Training children with autism spectrum disorder to undergo oral assessment using a digital iPad ® application

**Additional file 5.** Dental Anxiety in children with autism spectrum disorder: understanding frequency and associated variables

**Additional file 6.** Sensory-adapted dental environment for the treatment of patients with autism spectrum disorder

## Data Availability

All in figure 1 or tables.

## References

[1] American Psychiatric Association [APA]. Diagnostic and Statistical Manual of Mental Disorders. 5th ed. Washington, DC: American Psychiatric Association (2013).

[2] Brickhouse TH, Farrington FH, Best AM, Ellsworth CW (2009). Barriers to dental care for children in Virginia with autism spectrum disorders. J Dent Child (Chic).

[3] Loo CY, Graham RM, Hughes CV (2008). The caries experience and behavior of dental patients with autism spectrum disorder. J Am Dent Assoc..

[4] Marshall J, Sheller B, Williams BJ, Mancl L, Cowan C (2007). Cooperation predictors for dental patients with autism. Pediatr Dent..

[5] Friedlander AH, Yagiela JA, Paterno VI, Mahler ME (2006). The neuropathology, medical management and dental implications of autism. J Am Dent Assoc..

[6] Delli K, Reichart PA, Bornstein MM, Livas C (2013). Management of children with autism spectrum disorder in the dental setting: concerns, behavioural approaches and recommendations. Med Oral Patol Oral Cir Bucal..

[7] Stein LI, Lane CJ, Williams ME (2014). Physiological and behavioral stress and anxiety in children with autism spectrum disorders during routine oral care. J Biomed Res Int.

[8] Marshall J, Sheller B, Mancl L (2010). Caries-risk assessment and caries status of children with autism. J Pediatr Dent.

[9] Sahab LA. Investigating Dental Anxiety in Individuals With Autism Spectrum Disorders. Ph.D. thesis. Reading: University of Reading (2017).

[10] Chistol LT, Bandini LG, Must A, Phillips S, Cermak SA, Curtin C (2018). Sensory sensitivity and food selectivity in children with autism spectrum disorder. J Autis Dev Disord..

[11] Elmore JL, Bruhn AM, Bobzien JL (2016). Interventions for the reduction of dental anxiety and corresponding behavioral defificits in children with autism spectrum disorder. Am Dental Hygien Associat..

[12] American Academy of Pediatric Dentistry (2016). Guideline on behavior guidance for the pediatric dental patient. Pediatr Dent..

[13] Lefer G, Rouches A, Bourdon P (2019). Training children with autism spectrum disorder to undergo oral assessment using a digital iPad application. J Eur Arch Paediatr Dent.

[14] Knight V, Sartini E, Spriggs AD (2015). Evaluating visual activity schedules as evidence-based practice for individuals with autism spectrum disorders J. J Autism Dev Disord..

[15] Isong IA, Rao SR, Holifield C (2014). Addressing dental fear in children with autism spectrum disorders: a randomized controlled pilot study using electronic screen media. J Clin Pediatr.

[16] Cermak SA, Stein Duker LI, Williams ME (2015). Sensory Adapted Dental Environments to Enhance Oral Care for Children with Autism Spectrum Disorders: A Randomized Controlled Pilot Stud J. J Autism Dev Disord.

[17] Shapiro M, Melmed RN, Sgan-Cohen HD (2009). Effect of sensory adaptation on anxiety of children with developmental disabilities: a new approach. J Pediatr Dent.

[18] Moher D, Shamseer L, Clarke M, Ghersi D, Liberati A, Petticrew M, Shekelle P, Stewart LA (2015). PRISMA-P Group. Preferred reporting items for systematic review and meta-analysis protocols (PRISMA-P) 2015 statement. Syst Rev.

[19] Stang A. Critical evaluation of the Newcastle-Ottawa scale for the assessment of the quality of nonrandomized studies in meta-analyses. Eur J Epidemiol. 2010;25(9):603–5. 10.1007/s10654-010-9491-z 20652370

[20] Rutter M (1985). The treatment of autistic children. J Child Psychol Psychiatry..

[21] Cohen DJ, Donnellan A (1987). Handbook of Autism and Pervasive Developmental Disorders.

[22] Park Y, Guzick AG, Schneider SC (2022). Dental anxiety in children with autism spectrum disorder: understanding frequency and associated variables [J]. Front Psychiatry.

[23] Marshall J, Sheller B, Williams BJ (2007). Cooperation predictors for dental patients with autism [J]. Pediatr Dent.

[24] Dawson ME, Schell AM, Rissling A (2010). Psychophysiological prodromal signs of schizophrenic relapse: a pilot study. J Schizophr Res.

[25] Hutchins TL, Prelock PA (2014). Using communication to reduce challenging behaviors in individuals with autism spectrum disorders and intellectual disability. J Child Adolesc Psychiatr Clin N Am.

[26] Pilebro C, Bäckman B (2005). Teaching oral hygiene to children with autism. Int J Paediatr Dent..

[27] Weil TN, Bagramian RA, Inglehart MR (2011). Treating patients with autism spectrum disorder-SCDA members’ attitudes and behavior. Spec Care Dentist.

[28] Orellana LM, Martínez-Sanchis S, Silvestre FJ (2014). Training adults and children with an autism spectrum disorder to be compliant with a clinical dental assessment using a TEACCH-based approach. J Autism Dev Disord..

[29] Fallea A, Zuccarello R, Roccella M, Quatrosi G, Donadio S, Vetri L, Calì F. Sensory-Adapted Dental Environment for the Treatment of Patients with Autism Spectrum Disorder. Children (Basel). 2022;9(3):393. 10.3390/children9030393 PMC8947452 35327765

