# Supplementary material for: Management strategies of dental anxiety and uncooperative behaviors in children with Autism spectrum disorder
Source: BMC Pediatr. 2023 Dec 4;23:612. doi: 10.1186/s12887-023-04439-7 (PMC10694959; doi:10.1186/s12887-023-04439-7)
Supplement: Supplementary file 1 — Additional file 1. Addressing dental fear in children with autism spectrum disorders: a randomized controlled pilot study using electronic screen media [file 12887_2023_4439_MOESM1_ESM.pdf]

Article

Clinical Pediatrics
2014, Vol. 53(3) 230–237
© The Author(s) 2014
Reprints and permissions:
sagepub.com/journalsPermissions.nav
DOI: 10.1177/0009922813517169
cpj.sagepub.com
SAGE

Addressing Dental Fear in Children With Autism Spectrum Disorders: A Randomized Controlled Pilot Study Using Electronic Screen Media

Inyang A. Isong, MD, MPH, SM[1,2], Sowmya R. Rao, PhD[3,4], Chloe Holifield[5], Dorothea Iannuzzi, LICSW, BCBA[6,7], Ellen Hanson, PhD[1], Janice Ware, PhD[1], and Linda P. Nelson, DMD, MScD[1,8]

Abstract

Background. Dental care is a significant unmet health care need for children with autism spectrum disorders (ASD). Many children with ASD do not receive dental care because of fear associated with dental procedures; oftentimes they require general anesthesia for regular dental procedures, placing them at risk of associated complications. Many children with ASD have a strong preference for visual stimuli, particularly electronic screen media. The use of visual teaching materials is a fundamental principle in designing educational programs for children with ASD. *Purpose*. To determine if an innovative strategy using 2 types of electronic screen media was feasible and beneficial in reducing fear and uncooperative behaviors in children with ASD undergoing dental visits. *Methods*. We conducted a randomized controlled trial at Boston Children's Hospital dental clinic. Eighty (80) children aged 7 to 17 years with a known diagnosis of ASD and history of dental fear were enrolled in the study. Each child completed 2 preventive dental visits that were scheduled 6 months apart (visit 1 and visit 2). After visit 1, subjects were randomly assigned to 1 of 4 groups: (1) group A, control (usual care); (2) group B, treatment (video peer modeling that involved watching a DVD recording of a typically developing child undergoing a dental visit); (3) group C, treatment (video goggles that involved watching a favorite movie during the dental visit using sunglass-style video eyewear); and (4) group D, treatment (video peer modeling plus video goggles). Subjects who refused or were unable to wear the goggles watched the movie using a handheld portable DVD player. During both visits, the subject's level of anxiety and behavior were measured using the Venham Anxiety and Behavior Scales. Analyses of variance and Fisher's exact tests compared baseline characteristics across groups. Using intention to treat approach, repeated measures analyses were employed to test whether the outcomes differed significantly: (1) between visits 1 and 2 within each group and (2) between each intervention group and the control group over time (an interaction). *Results*. Between visits 1 and 2, mean anxiety and behavior scores decreased significantly by 0.8 points (P = .03) for subjects within groups C and D. Significant changes were not observed within groups A and B. Mean anxiety and behavior scores did not differ significantly between groups over time, although group A versus C pairwise comparisons showed a trend toward significance (P = .06). *Conclusion*. These findings suggest that certain electronic screen media technologies may be useful tools for reducing fear and uncooperative behaviors among children with ASD undergoing dental visits. Further studies are needed to assess the efficacy of these strategies using larger sample sizes. Findings from future studies could be relevant for nondental providers who care for children with ASD in other medical settings.

[1]Boston Children's Hospital, Boston, MA, USA
[2]Harvard Medical School, Boston, MA, USA
[3]University of Massachusetts Medical School, Worcester, MA, USA
[4]Center for Health Quality, Outcomes, and Economic Research, Bedford VA, MA, USA
[5]Masachusetts General Hospital, Boston, MA, USA
[6]Harvard Vanguard Medical Associates, Concord, MA, USA
[7]Simmons College School of Social Work, Boston, MA, USA
[8]Harvard School of Dental Medicine, Boston, MA, USA

Corresponding Author:
Inyang A. Isong, Children's Hospital Primary Care Center, Boston Children's Hospital, 300 Longwood Avenue, Boston, MA 02115, USA.
Email: inyang.isong@childrens.harvard.edu

Downloaded from cpj.sagepub.com at TEMPLE UNIV on April 22, 2015

Keywords
children, autism spectrum disorders, electronic screen media, dental fear

Introduction

Dental care is the most common unmet health care need for children with special health care needs in the United States.[1,2] Children with autism spectrum disorders (ASD) are a group of children with special health care needs frequently cited as having difficulties complying with dental care procedures.[3-9] There is also a higher prevalence of comorbidities like anxiety among children with ASD.[10] ASD may be associated with unusual responses to sensory stimuli, such as oversensitivity to unfamiliar sounds, touch, and heightened reactions to light.[11] These special characteristics often impede the children's ability to cooperate in dental settings.[9]

To minimize dental fear among typically developing children, pediatric dentists use behavior guidance techniques that are tailored to the individual patient and the practitioner. The techniques must "promote communication, alleviate fear and anxiety, deliver quality dental care, build a trusting relationship between dentist, child and parent, and promote the child's positive attitude toward oral/dental health and oral health care."[12] The American Academy of Pediatric Dentistry–recommended nonpharmacologic techniques[13-19] for children include the following: tell–show–do, voice control, nonverbal communication, positive reinforcement, and distraction. However, these behavior guidance techniques used for typically developing children with dental fear are generally ineffective for children with autism.[20,21] Children with autism have a higher prevalence of poor oral hygiene status, dental caries, and periodontal disease.[22,23] Furthermore, children with autism often require sedation or general anesthesia for routine dental procedures,[6-8,20] placing them at risk of associated complications.

Children with ASD often have a strong preference for taking information using visual input, particularly electronic screen media such as television and videos/DVDs.[24-28] The preference for electronic screen media has been successfully exploited in educational and therapeutic settings to engage and motivate children with ASD in learning activities.[25-27] However, few studies have examined the potential of media technology to improve the dental visit experience for children with autism. One study examined the feasibility of video peer modeling for children with ASD in a dental setting.[6] The study was not a randomized controlled trial and had a limited sample of 3 subjects. The effectiveness of video peer modeling or other types of electronic screen media interventions in reducing dental fear among a larger sample of children with ASD has not been evaluated. The goal of this study was to determine if electronic screen media strategies are effective and practical in the dental office setting for reducing fear and increasing compliance during dental procedures.

Methods

We conducted a pilot randomized controlled trial at Boston Children's Hospital (BCH) dental clinic between December 2010 and July 2012. The study protocol was approved by the BCH Institutional Review Board.

Subjects

Eighty (80) subjects aged 7 to 17 years with a known diagnosis of autism were enrolled in the study. Subjects were recruited from the BCH dental clinic and from an ongoing autism study ("Phenotypic and Genetic Factors in Autism Spectrum Disorders") that was being conducted within the BCH Developmental Medicine Center. Eligibility criteria were (1) child who receives care in the BCH dental clinic with a diagnosis of ASD as documented in the electronic medical records, or a child who was referred from the autism data registry project and (2) child with a history of dental fear, per parental report. Exclusion criteria included (1) children with motor handicaps (eg, cerebral palsy), and significant neurosensory impairments, including vision and hearing losses that would limit the ability to use intervention materials; (2) children with parents who did not speak English or Spanish; and (3) children who presented to clinic visits without the support of parents or legal guardians. A list of potentially eligible children was generated from the dental clinic patient records, and from a list of families participating in the autism data registry project who consented to contact for other studies related to ASD. An introductory letter was mailed and followed by a telephone follow-up call for those who did not return the enclosed opt-out postcard. Subject recruitment was initially slow. The target enrollment population was expanded to include children who met eligibility criteria who were in the clinic waiting area before a dental visit. Written informed consent and/or subject assent (if appropriate) was obtained prior to initiation of study procedures.

Intervention

Study data were collected only during preventive dental visits in order to maintain uniformity across visits. A preventive dental visit is a routine appointment that occurs every 6 months. The visits involve extra-oral and intraoral examination, scaling (if needed), prophylaxis, application of fluoride varnish and periodically, radiographs.

Downloaded from cpj.sagepub.com at TEMPLE UNIV on April 22, 2015

All visits were scheduled with BCH dentists, including faculty and residents. Enrolled subjects initially completed a baseline preventive visit (visit 1), followed by a follow-up preventive visit 4 to 6 months later (visit 2). All subjects were assigned to 1 of the following 4 groups using a SAS-generated randomization scheme (1:1:1:1 equal ratio within each group): (1) control group (group A); (2) video peer modeling only group (group B); (3) video goggles only group (group C); and (4) video peer modeling plus video goggles group (group D). Subjects who refused or were unable to wear the goggles watched the movie using a handheld portable DVD player.

Definitions

Video Peer Modeling. A DVD recording was filmed of a typically developing child undergoing a preventive dental visit at the actual treatment site. The video demonstrates various stages of the dental visit experience, including arriving at BCH, checking in at the dental clinic reception desk, receiving care in the dental chair (cleaning, scaling, polishing, and fluoride varnish application), and obtaining a dental radiograph. Children assigned to intervention groups B or D were mailed a copy of the peer modeling DVD at least 4 weeks before the scheduled return visit. Parents were asked to review the video with their child at least once, but as many times as possible. Each child also watched the DVD in the waiting room, 15 minutes before the return dental visit.

Video Goggles. Sunglass-style video eyewear (Vuzix Corporation, Rochester, NY) was used that contains 2 computer screens for viewing 2D or 3D movies. Subjects randomized to group C or D watched a 2D movie during the return visit, using the video goggles.

Children assigned to group A (control group) received a preventive dental visit (usual care) and therefore had no intervention before or during the return visit. Parents of subjects assigned to this group were not provided any instructions specific to this study prior to visit 2.

Data Collection

Primary Outcome. The subject's level of anxiety was the primary outcome. Anxiety was assessed at the end of the dental visit, using the Venham Anxiety Rating Scale,[29] an instrument that assigns numerical values to observable behaviors associated with anxiety. It consists of 5 behaviorally defined categories ranked by severity.

Secondary Outcomes

a. *Behavior*: We assessed the subject's behavior at the end of the dental visit, using the Venham Behavior Rating Scale.[29,30] The scale consists of 5 behaviorally defined categories ranking severity on a scale from 0 to 5. Higher scores indicate greater levels of uncooperative behavior.[29,30]
b. *Physiological arousal*: The child's pulse rate was recorded pre and post visit 2 (follow-up visit) as an objective measure of physiological arousal during the dental procedure.

The Venham Anxiety and Behavior Rating Scales have been used extensively to assess the level of anxiety and behavior of children in dental settings.[31-35] Behavior and anxiety were measured during visit 1 and visit 2 for study subjects. The dentist and research assistant completed the Venham Anxiety and Behavioral Scale separately at the end of each visit, and the average of the 2 scores was recorded. During visit 2, the research assistant measured previsit (just after the child sat in the dental chair) and postvisit (after completion of the dental visit) pulse rates, using a pulse oximeter and finger probe. Although most patients tolerated this noninvasive procedure to measure pulse rates, several subjects became agitated. As such, we were unable to obtain measurements on all subjects.

Statistical Methods

Baseline data were collected for all 80 subjects at visit 1; 3 subjects dropped out and 8 did not return for visit 2. We analyzed all subjects in their assigned randomized group, consistent with an intention-to treat approach. We used analysis of variance (for continuous variables) and Fisher's exact test (for categorical variables) to compare baseline characteristics across the 4 groups. We employed repeated-measures analyses to test whether the Venham Anxiety Score and Venham Behavior Score (1) differed significantly between visits 1 and 2 within each group and (2) differed significantly between each intervention group and the control group over time. We conducted similar analyses comparing pre- and postvisit pulse rates for visit 2. The repeated-measures analysis was performed using PROC MIXED, with group, time, and a group × time interaction term included in the model. The differences between time points within each group were tested with the "ESTIMATE" statement and the pairwise differences between groups were tested with the "CONTRAST" statement. We performed all analyses using SAS software (Version 9.2; SAS Institute, Inc, Cary, NC).

Results

Figure 1 depicts the flow of subjects through the randomization, follow-up and analysis process. Overall, 80 children ranging in age from 7 to 17 years participated in

Downloaded from cpj.sagepub.com at TEMPLE UNIV on April 22, 2015

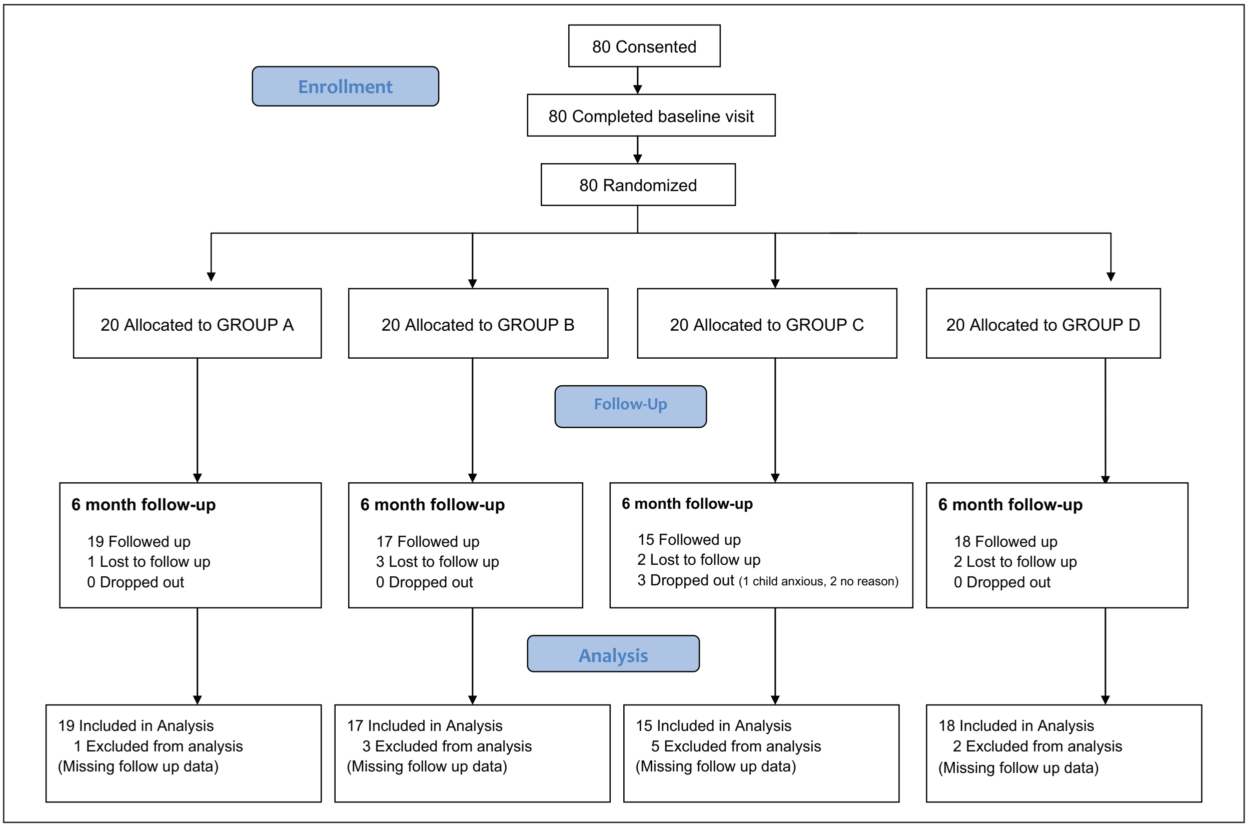

Figure 1. Flow of subjects through the randomized controlled trial. Group A = control group; group B = video peer modeling only group; group C = video goggles/DVD only group; group D = video peer modeling plus video goggles/DVD.

the study. The sample included 81% males with an average age of 9.9 years (SD = 2.44). Sixty percent (60%) of subjects' parents had at least a college degree, and race/ethnicity as follows: 53% Caucasian, 16% African American, 9% Asian, 4% Hispanic, and 16% other. There were no significant differences in the baseline characteristics of study subjects across the intervention and control groups. (Table 1) Overall, 20 subjects (8 in group B and 12 in group D) watched the peer modeling video more than once.

We also obtained subjects' Full Scale Intelligence Quotient (categorized as <50, 50-70, and >70), and verbal/nonverbal status from the medical records. We created a composite variable as an indicator of cognitive ability (moderate to severe intellectual disability, intellectual disability, and average/above average intelligence). We did not find any statistically significant differences across groups in subjects' cognitive ability ($P = .79$; data not shown).

Table 2 shows the mean Venham Anxiety and Behavior scores at baseline (visit 1) and follow-up (visit 2), as well as differences in visit 2 pre/post pulse rates, for subjects by randomization groups. Between visits 1 and 2, mean anxiety and behavior scores decreased significantly by 0.9 points ($P = .03$) for subjects within group C (video goggles or handheld DVD player only) and by 0.8 points ($P = .03$) for subjects within group D (video peer modeling plus video goggles or handheld DVD player). Mean anxiety and behavior scores did not change significantly for subjects within group A (control) and group B (video peer modeling only). In addition, mean anxiety and behavior scores did not differ significantly between each intervention group and the control group over time, although group A versus C pairwise comparisons showed a trend toward significance ($P = .06$; Table 3). Differences in pre/post pulse rates were not significant within or across groups.

We evaluated whether there were differences in outcomes depending on the number of times subjects in groups B or D watched the peer modeling video (1 vs >1), or if subjects in groups C or D used video goggles or a handheld DVD player. Our results indicated that mean anxiety and behavior scores were lower among subjects in group D who watched the peer modeling video more than once. Similarly, mean anxiety and behavior scores were lower among subjects in groups C

Downloaded from cpj.sagepub.com at TEMPLE UNIV on April 22, 2015

Table 1. Baseline Characteristics of Subjects, by Intervention and Control Groups.[a]

	Group A (n = 20)	Group B (n = 20)	Group C (n = 20)	Group D (n = 20)	P^b
Age in years, mean ± SD	10.2 ± 2.4	9.8 ± 2.2	9.7 ± 3.2	9.8 ± 2.1	.92
Male gender, n (%)	17 (85.0)	17 (85.0)	14 (70.0)	17 (85.0)	.62
Insurance, n (%)					.77
Public	7 (35.0)	8 (40.0)	8 (40.0)	10 (50.0)	
Private	13 (65.0)	8 (40.0)	12 (60.0)	10 (50.0)	
Parent education, n (%)					.18
<4-year college graduate	9 (45.0)	7 (35.0)	2 (10.0)	7 (35.0)	
≥4-year college graduate	9 (45.0)	9 (45.0)	11 (55.0)	9 (45.0)	
Treating dentist, n (%)					.09
Faculty	17 (85.0)	17 (85.0)	13 (65.0)	19 (95.0)	
Resident	3 (15.0)	3 (15.0)	7 (35.0)	1 (5.0)	

[a]Group A = control group; group B = video peer modeling only group; group C = video goggles (or portable DVD player) only group; group D = video peer modeling plus video goggles (or portable DVD player) group.
[b]Obtained from *t* tests or Fisher's exact tests.

Table 2. Pulse Rate, Venham Anxiety and Behavior Scores at Baseline (Visit 1) and Follow-up (Visit 2), by Randomization Groups.

Outcomes	Group A	Group B	Group C	Group D	P^a
Venham Anxiety, mean ± SD					.23
Visit 1	2.4 ± 1.8	2.6 ± 1.8	2.6 ± 1.3	2.9 ± 1.3	
Visit 2	2.3 ± 1.6	2.6 ± 1.9	1.7 ± 1.8	2.1 ± 1.6	
P^b	.64	.98	.03	.03	
Venham Behavior, mean ± SD					.07
Visit 1	2.2 ± 1.9	2.7 ± 1.8	2.5 ± 1.6	2.9 ± 1.5	
Visit 2	2.3 ± 1.6	2.9 ± 2.0	1.7 ± 1.9	2.1 ± 1.6	
P^b	.84	.69	.02	.03	
Pulse rate difference,[c] mean ± SD					.57
Visit 2	−1.8 ± 19.3	6.7 ± 9.2	2.0 ± 11.1	−2.8 ± 20.8	
P^b	.73	.30	.77	.40	

[a]Obtained from repeated-measures regression models. *P* values indicate whether the outcomes differ significantly by groups over time.
[b]Obtained from repeated-measures regression models. *P* values indicate whether the outcomes changed significantly over time for the particular group.
[c]Difference between pulse rate pre and post visit 2.

and D who wore the goggles, versus those who used the handheld DVD player. However, we were unable to test the statistical significance of these differences because of small sample sizes.

Discussion

The results of this pilot study suggest that certain electronic screen media may be feasible and useful strategies for addressing fear and uncooperative behaviors among children with ASD undergoing dental visits. For many children with autism, going to the dentist is a stressful event.[36,37] Parents sometimes avoid or postpone dental care for children with ASD because of the child's fear of the dental office visits and procedures.[36,37] The positive use of electronic screen media to teach children with ASD more adaptive behaviors has been documented in prior research.[38] Our study sought to harness the potential for behavior change during the dental visit. The study results found that mean anxiety and behavior scores among children randomized to watch a movie during the dental visit, using either video goggles or a portable DVD player, decreased significantly between the baseline and follow-up visit. Watching a video with the video goggles or DVD player could have served as a distraction, thereby reducing associated anxiety and resultant uncooperative behaviors. According to McCaul and Malott,[39] distraction works by diverting attention

Downloaded from cpj.sagepub.com at TEMPLE UNIV on April 22, 2015

Table 3. *P* Values for Pairwise Comparisons of Mean Venham Anxiety and Behavior Scores for Control and Intervention Groups.

	P Values	
Pairwise Comparisons	Mean Venham Anxiety Scores	Mean Venham Behavior Scores
Group A and group B	.73	.88
Group A and group C	.17	.06
Group A and group D	.21	.08

from noxious stimuli toward more pleasant ones. Distraction through electronic screen media has been used as a cognitive–behavioral intervention to address anxiety in other research.[40,41] One study using virtual reality technology as a distraction demonstrated decreased anxiety in children with cancer receiving invasive medical procedures.[40] Although mean scores for children randomized to the video goggles (or portable DVD player) plus video peer modeling group (Group D) decreased significantly between visits, scores did not improve for children in the video peer modeling only group. As such, it is possible that significant changes among children in Group D were a result of the video goggles (or portable DVD player), rather than the video peer modeling.

Video peer modeling is an approach used to teach new skills by watching a video model of the desired behavior during a demonstration of a specific task. The method has been used in other research to teach children with ASD various social and play skills.[42,43] The approach is based on applied behavioral analytic principles, an evidence-based treatment for autistic behaviors. In the dental setting, video peer modeling has the potential to desensitize children with autism to the dental visit experience. A small, nonrandomized study by Luscre and Center[6] demonstrated that video peer modeling could be used to train children with ASD to cooperate during a dental exam. However, the study design limited the validity and generalizability of its findings.

Study results also found that the mean anxiety and behavior scores did not differ significantly between groups over time, although pairwise comparisons between children randomized to group A (control) versus group C (video goggles or handheld DVD player only) showed a trend toward significance. This finding suggests that certain kinds of electronic screen media, when used as a distraction during the visit, could potentially reduce uncooperative behaviors in children with autism undergoing dental procedures. Further studies are needed to assess the efficacy of these strategies using larger sample sizes.

This study had several limitations. This was not a blinded study, thus, the raters were aware of the treatment assignment of the subjects. As such, their scoring for subjects' anxiety and behavior could have been biased, thereby influencing results. However, given the nature of the study, blinding was not feasible. The dose of exposure to the peer video varied across subjects, ranging from 1 to 5 viewings. All children watched the video at least once, just before the follow-up dental visit. Although our findings indicated that subjects in group D who watched the peer modeling video more than once had lower mean scores, small sample sizes precluded testing for statistical significance of this interaction. Because some children with autism have heightened responses to sensory input, some of the study procedures may have contributed toward some subjects' anxiety. For example, subjects' pulse rate measurements were obtained using finger probes in order to have an objective measure of physiological arousal. This is a relatively noninvasive procedure. However, it made a few subjects anxious. Although many subjects really enjoyed using the video goggles, others who tried on the video goggles could also have become more anxious as a result. In future studies, researchers should strive to minimize procedures that could increase anxiety. A sensory or preference assessment could be done prior to the start of the study to address this; other handheld devices like iPads could also be considered as alternatives. It is possible that effect of electronic screen media could have been modified by the subjects' level of intellectual functioning but we were unable to assess this because of small sample sizes. Finally, the pilot nature of this study did not provide adequate power to detect significant differences across intervention and control groups.

Conclusion

The results of this pilot study demonstrated the feasibility of using electronic screen media to address dental fear in children with autism. The results suggest that certain electronic screen media technologies may be useful tools for reducing fear and uncooperative behaviors among children with ASD undergoing dental visits. Further studies are needed to assess the efficacy of these strategies using larger sample sizes. Findings from future studies could be relevant for nondental providers who care for children with ASD in other medical settings.

Downloaded from cpj.sagepub.com at TEMPLE UNIV on April 22, 2015

Authors' Note

This study is registered on clinicaltrials.gov: ClinicalTrials.gov identifier: NCT01784276.

Acknowledgments

We would like to thank the staff of the Boston Children's Hospital dental clinic, New England Center for Children, and the Massachusetts Institute of Technology Media Lab for their assistance on this project.

Declaration of Conflicting Interests

The author(s) declared no potential conflicts of interest with respect to the research, authorship, and/or publication of this article.

Funding

The author(s) disclosed receipt of the following financial support for the research, authorship, and/or publication of this article: This project was supported by an award from the Deborah Munroe Noonan Memorial Research Fund.

References

1. Lewis C, Robertson AS, Phelps S. Unmet dental care needs among children with special health care needs: implications for the medical home. *Pediatrics*. 2005;116:e426-e431.
2. Newacheck PW, Hughes DC, Hung YY, Wong S, Stoddard JJ. The unmet health needs of America's children. *Pediatrics*. 2000;105(4 pt 2):989-997.
3. Kamen S, Skier J. Dental management of the autistic child. *Spec Care Dentist*. 1985;5:20-23.
4. Klein U, Nowak AJ. Characteristics of patients with autistic disorder (AD) presenting for dental treatment: a survey and chart review. *Spec Care Dentist*. 1999;19:200-207.
5. Davila JM, Jensen OE. Behavioral and pharmacological dental management of a patient with autism. *Spec Care Dentist*. 1988;8:58-60.
6. Luscre DM, Center DB. Procedures for reducing dental fear in children with autism. *J Autism Dev Disord*. 1996;26:547-556.
7. Fahlvik-Planefeldt C, Herrström P. Dental care of autistic children within the non-specialized public dental service. *Swed Dent J*. 2001;25:113-118.
8. Lowe O, Lindemann R. Assessment of the autistic patient's dental needs and ability to undergo dental examination. *ASDC J Dent Child*. 1985;52:29-35.
9. Brickhouse TH, Farrington FH, Best AM, Ellsworth CW. Barriers to dental care for children in Virginia with autism spectrum disorders. *J Dent Child (Chic)*. 2009;76:188-193.
10. Kim JA, Szatmari P, Bryson SE, Streiner DL, Wilson FJ. The prevalence of anxiety and mood problems among children with autism and Asperger syndrome. *Autism*. 2000;4:117-132.
11. American Psychiatric Association. Task Force on DSM-IV, ed. *Diagnostic and Statistical Manual of Mental Disorders: DSM-IV-TR*. 4th ed. Washington, DC: American Psychiatric Association; 2000.
12. American Academy of Pediatric Dentistry. Guideline on behavior guidance for the pediatric dental patient. *Pediatr Dent*. 2012;34:170-182.
13. Pinkham JR. Patient management. in: Pinkham JR, Casamassimo PS, Fields HW Jr, McTigue DJ, Nowak AJ, eds. *Pediatric Dentistry: Infancy Through Adolescence*. 4th ed. St Louis, MO: Elsevier Saunders; 2005:394-413.
14. Law CS, Blain S. Approaching the pediatric dental patient: a review of non-pharmacologic behavior management strategies. *J Calif Dent Assoc* 2003;31:703-713.
15. Feigal RJ. Guiding and managing the child dental patient: a fresh look at old pedagogy. *J Dent Educ*. 2001;65:1369-1377.
16. Eaton JJ, McTigue DJ, Fields HW Jr, Beck M. Attitudes of contemporary parents towards behavior management techniques used in pediatric dentistry. *Pediatr Dent*. 2005;27:107-113.
17. Wright GZ, Stiger JI. Non-pharmacologic management of children's behaviors. In Dean JA, Avery DR, McDonald RE, eds. *McDonald and Avery's Dentistry for the Child and Adolescent*. 9th ed. Maryland Heights, MO: Mosby Elsevier; 2011:27-40.
18. Chambers DW. Communicating with the young dental patient. *J Am Dent Assoc*. 1976;93:793-799.
19. Pinkham JR. The roles of requests and promises in child management. *J Dent Child*. 1993;60:169-174.
20. Marshall J, Sheller B, Williams BJ, Mancl L, Cowan C. Cooperation predictors for dental patients with autism. *Pediatr Dent*. 2007;29:369-376.
21. Loo CY, Graham RM, Hughes CV. Behaviour guidance in dental treatment of patients with autism spectrum disorder. *Int J Paediatr Dent*. 2009;19:390-398.
22. Butts J. Dental status of mentally retarded children: II. A survey of the prevalence of certain dental conditions in mentally retarded children of Georgia. *J Public Health Dent*. 1967;27:195-211.
23. Starks D., Market G., Miller C, Greenbaum J. Day to day dental care: a parent's guide. *Exceptional Parent*. 1985:13-17.
24. Shane HC, Albert PD. Electronic screen media for persons with autism spectrum disorders: results of a survey. *J Autism Dev Disord*. 2008;38:1499-1508.
25. Bernard-Opitz V, Sriram N, Nakhoda-Sapuan S. Enhancing social problem solving in children with autism and normal children through computer-assisted instruction. *J Autism Dev Disord*. 2001;31:377-384.
26. LeBlanc LA, Coates AM, Daneshvar S, Charlop-Christy MH, Morris C, Lancaster BM. Using video modeling and reinforcement to teach perspective-taking skills to children with autism. *J Appl Behav Anal*. 2003;36:253-257.
27. Charlop-Christy MH, Le L, Freeman KA. A comparison of video modeling with in vivo modeling for teaching

Downloaded from cpj.sagepub.com at TEMPLE UNIV on April 22, 2015

children with autism. *J Autism Dev Disord*. 2000;30:537-552.
28. Moore M, Calvert S. Brief report: vocabulary acquisition for children with autism: teacher or computer instruction. *J Autism Dev Disord*. 2000;30:359-362.
29. Venham LL, Gaulin-Kremer E, Munster E, Bengston-Audia D, Cohan J. Interval rating scales for children's dental anxiety and uncooperative behavior. *Pediatr Dent*. 1980;2:195-202.
30. Newton JT, Buck DJ. Anxiety and pain measures in dentistry: a guide to their quality and application. *J Am Dent Assoc*. 2000;131:1449-1457.
31. Venham LL, Goldstein M, Gaulin-Kremer E, Peteros K, Cohan J, Fairbanks J. Effectiveness of a distraction technique in managing young dental patients. *Pediatr Dent*. 1981;3:7-11.
32. Alwin NP, Murray JJ, Britton PG. An assessment of dental anxiety in children. *Br Dent J*. 1991;171:201-207.
33. Nathan JE, Venham LL, West MS, Werboff J. The effects of nitrous oxide on anxious young pediatric patients across sequential visits: a double-blind study. *ASDC J Dent Child*. 1988;55:220-230.
34. Prabhakar AR, Marwah N, Raju OS. A comparison between audio and audiovisual distraction techniques in managing anxious pediatric dental patients. *J Indian Soc Pedod Prev Dent*. 2007;25:177-182.
35. Marwah N, Prabhakar AR, Raju OS. Music distraction—its efficacy in management of anxious pediatric dental patients. *J Indian Soc Pedod Prev Dent*. 2005;23:168-170.
36. Howlin P, Rutter M, eds. *Treatment of Autistic Children*. Chichester, England: Wiley; 1987.
37. Cohen DJ, Donnellan A, eds. *Handbook of Autism and Pervasive Developmental Disorders*. Chichester, England: Wiley; 1987.
38. Mineo BA, Ziegler W, Gill S, Salkin D. Engagement with electronic screen media among students with autism spectrum disorders. *J Autism Dev Disord*. 2009;39:172-187.
39. McCaul KD, Malott JM. Distraction and coping with pain. *Psychol Bull*. 1984;95:516-533.
40. Gershon J, Zimand E, Pickering M, Rothbaum BO, Hodges L. A pilot and feasibility study of virtual reality as a distraction for children with cancer. *J Am Acad Child Adolesc Psychiatry*. 2004;43:1243-1249.
41. Schneider SM, Workman ML. Virtual reality as a distraction intervention for older children receiving chemotherapy. *Pediatr Nurs*. 2000;26:593-597.
42. Wert BY, Neisworth JT. Effects of video self-modeling on spontaneous requesting in children with autism. *J Positive Behav Intervent*. 2003;5:30-34.
43. D'Ateno P, Mangiapanello K, Taylor B. Using video modeling to teach complex play sequences to a preschooler with autism. *J Positive Behav Intervent*, 2003;5:5-11.

Downloaded from cpj.sagepub.com at TEMPLE UNIV on April 22, 2015
