# Supplementary material for: Management strategies of dental anxiety and uncooperative behaviors in children with Autism spectrum disorder
Source: BMC Pediatr. 2023 Dec 4;23:612. doi: 10.1186/s12887-023-04439-7 (PMC10694959; doi:10.1186/s12887-023-04439-7)
Supplement: Supplementary file 3 — Additional file 3. Sensory adapted dental environments to enhance oral care for children with autism spectrum disorders: a randomized controlled pilot study [file 12887_2023_4439_MOESM3_ESM.pdf]

J Autism Dev Disord
DOI 10.1007/s10803-015-2450-5

ORIGINAL PAPER

Sensory Adapted Dental Environments to Enhance Oral Care for Children with Autism Spectrum Disorders: A Randomized Controlled Pilot Study

Sharon A. Cermak[1,2] · Leah I. Stein Duker[1] · Marian E. Williams[3] · Michael E. Dawson[4] · Christianne J. Lane[5] · José C. Polido[6]

© Springer Science+Business Media New York 2015

Abstract This pilot and feasibility study examined the impact of a sensory adapted dental environment (SADE) to reduce distress, sensory discomfort, and perception of pain during oral prophylaxis for children with autism spectrum disorder (ASD). Participants were 44 children ages 6–12 (n = 22 typical, n = 22 ASD). In an experimental cross-over design, each participant underwent two professional dental cleanings, one in a regular dental environment (RDE) and one in a SADE, administered in a randomized and counterbalanced order 3–4 months apart. Outcomes included measures of physiological anxiety, behavioral distress, pain intensity, and sensory discomfort. Both groups exhibited decreased physiological anxiety and reported lower pain and sensory discomfort in the SADE condition compared to RDE, indicating a beneficial effect of the SADE.

Keywords Autism spectrum disorder · Electrodermal activity · Skin conductance · Sensory processing · Oral health · Occupational therapy · Dental anxiety

Introduction

Oral care is integral for both psychological and physiological health and well-being (U.S. Department of Health and Human Services 2010). Many children with special needs have poorer oral health than typically developing children and experience difficulty obtaining adequate oral health care (Brickhouse et al. 2009; Kopycka-Kedzierawski and Auinger 2008; Nelson et al. 2011). Sometimes the cause is lack of access, and other times lack of child cooperation. For children with autism spectrum disorder who exhibit impaired social interaction, social

✉ Leah I. Stein Duker
LStein@usc.edu

Sharon A. Cermak
Cermak@usc.edu

Marian E. Williams
MWilliams@chla.usc.edu

Michael E. Dawson
MDawson@usc.edu

Christianne J. Lane
Christianne.Lane@med.usc.edu

José C. Polido
JPolido@chla.usc.edu

[1] Division of Occupational Science and Occupational Therapy, Herman Ostrow School of Dentistry, University of Southern California, 1540 Alcazar St, CHP 133, Los Angeles, CA 90089, USA

[2] Department of Pediatrics, USC Keck School of Medicine, University of Southern California, 1540 Alcazar St, CHP 133, Los Angeles, CA 90089, USA

[3] Keck School of Medicine of USC, University of Southern California University Center for Excellence in Developmental Disabilities (USC UCEDD), Children's Hospital Los Angeles, 4650 Sunset Blvd, MS#53, Los Angeles, CA 90027, USA

[4] Department of Psychology of the Dana and David Dornsife College of Letters, Arts and Sciences, University of Southern California, Los Angeles, CA 90027, USA

[5] Division of Biostatistics, Department of Preventive Medicine, University of Southern California, 2001 N. Soto St. SSB 220X, Los Angeles, CA 90089, USA

[6] Children's Hospital Los Angeles, Herman Ostrow School of Dentistry, University of Southern California, 4650 Sunset Blvd, MS#116, Los Angeles, CA 90027, USA

Published online: 01 May 2015

communication, and restricted and repetitive patterns of behavior, interests, or activities (APA 2013), it is often both. Children's lack of cooperation may be compounded by the reported inadequate training received by dentists to address such challenges, leading to many dental practitioners being unwilling to treat children with ASD (Casamassimo et al. 2004; Dao et al. 2005; Nelson et al. 2011; Weil and Inglehart 2010).

Difficulty with sensory processing is a well-recognized feature in autism, with reports indicating the presence of some form of sensory processing difficulty in up to 95 % of children with ASD (Baker et al. 2008; Baranek et al. 2006; Ben-Sasson et al. 2009b; Geschwind 2009; Tomchek and Dunn 2007). Research by Stein et al. (2011, 2012b, 2013) suggests that sensory over-responsivity is an important factor influencing the ability of children with ASD to receive oral care, with a relationship existing between sensory over-responsivity and uncooperative behaviors in the dental office. Sensory stimuli encountered in the dental office, such as bright fluorescent lights, touch in and around the mouth, as well as the taste and smell of oral care products, have the potential to negatively impact these children, increasing their negative responses and making it more difficult for dentists to provide treatment.

In a recent survey completed by parents of 196 children with ASD and 202 typically developing (TD) children, 60 % of parents of children with ASD reported moderate to extreme difficulty having the dentist or hygienist clean their child's teeth, in comparison to 13 % of parents of TD children (Stein et al. 2012a). Compared to parents of TD children, significantly more parents of children with ASD reported that their child was moderately to very over-responsive to sensory stimuli across all sensory modalities (touch, oral, taste, smell, sound, vibration, movement, light). Using cut scores to identify children with ASD who were "over-responders" to sensory stimuli, 65 % of parents of such children reported that it was moderately to extremely difficult to have the dentist/hygienist clean their child's teeth, in comparison to only 39 % of children with ASD not categorized as over-responders (Stein et al. 2013). Likewise, significantly more parents of children with ASD who were over-responders, in comparison to other children with ASD who did not exhibit sensory over-responsivity, reported that their child's uncooperative behaviors increased at the dental office (52 vs. 34 %) and that their child required restraint often or almost always for routine dental cleanings (38 vs. 18 %; Stein et al. 2013). These findings support a key premise of the current study, namely that sensory over-responsivity is associated with both difficulty with dental cleanings and increased uncooperative behaviors during dental care.

Because the prevalence of ASD has risen dramatically in recent years (CDC 2014), now 1 in 68 children, and because oral health is essential to overall health and well-being (U.S. HHS 2010), it is important to identify innovative solutions that enable dentists to more readily perform standard clinic-based procedures for these children. Shapiro et al. (2009a, b) conducted a study that modified the sensory characteristics of the dental office for children with developmental and intellectual disabilities (not ASD) and found decreased physiological anxiety and behavioral distress. Since sensory issues are so common in children with ASD, we believe that an intervention of this nature would be of benefit for children with ASD.

The purpose of this pilot study was to gather preliminary data on the feasibility and efficacy of a sensory adapted dental environment (SADE), as compared to a regular dental environment (RDE), to reduce the sensory-aversive characteristics of the environment, thereby decreasing children's arousal, uncooperative behavior, pain, and sensory discomfort. We hypothesized that (1) children with ASD would exhibit more behavioral and physiological distress, pain, and sensory discomfort during dental cleanings in both conditions than TD children; (2) children with ASD and, to a lesser extent, TD children would exhibit less behavioral and physiological distress, pain, and sensory discomfort during cleanings in the SADE environment as compared to the RDE; and (3) the SADE environment would be more cost-effective as evidenced by a shorter duration for dental cleaning, fewer staff, and reduced need for anesthesia in the SADE condition as compared to the RDE. This paper presents the results of the intervention's efficacy on key measures of behavioral and physiological distress, pain, sensory discomfort, and cost.

Methods

Design

Using an experimental randomized crossover design, we examined behavioral and physiological distress, pain, and sensory discomfort of children with ASD and TD children during routine professional dental cleanings in two conditions, a RDE and a SADE, presented in a counterbalanced manner (see Fig. 1) 3–4 months apart.

Participants

Participants included 22 children with autism spectrum disorder (ASD) and 22 typically developing children (TD), aged 6 through 12 years. Eligibility criteria for both groups included English or Spanish speaking parents, children who had at least one prior oral cleaning but did not have a

cleaning within the previous 4–6 months and who did not have significant motor impairments or genetic, endocrine, or metabolic dysfunction. TD children with a sibling with ASD or with an identified psychological disorder such as ADHD, bipolar disorder, or anxiety disorder were excluded. Children with ASD were required to have a confirmed ASD diagnosis using the Autism Diagnostic Observation Schedule (Lord et al. 1999), which was provided as part of the study procedures if not previously completed.

Participant and family characteristics for ASD and TD groups are reported in Table 1. There were no statistically significant between group differences in age, ethnicity, race, or parental education. The groups differed for gender, with the ASD group having significantly more male participants (as expected based on disease prevalence; CDC 2014). The ASD group had significantly lower average expressive language scores on the Vineland ($M \pm SD = 9.0 \pm 3.2$) than the TD group (15.5 ± 2.6; $p < 0.001$), with the majority of ASD participants scoring below the 18th percentile (low to moderately low range; 91 %). Compared to parents of TD children, parents of children with ASD also rated their child as having significantly higher levels of sensory processing difficulty, general anxiety, and dental anxiety (all p's < 0.001; Table 1).

Procedures

Participants for both groups were recruited from the Dental Clinic in a large urban children's hospital, which provides services to an ethnically diverse (60 % Hispanic; 5 % Asian, 16 % African American) and low income (90 %) under-served population. As illustrated in Fig. 2, records for upcoming scheduled participants were reviewed for initial eligibility. Those who met criteria were contacted by phone and the study was explained to them in their native language, either English or Spanish. If parents expressed interest in learning more about the study, a visit was scheduled to further explain the details of the study, obtain consent, and introduce children to the electrodes used for the study. For children with ASD, parents were asked to provide written documentation of ADOS results. Written reports documenting ADOS results were available for 8 participants; the other 14 participants had a separate visit scheduled to administer the ADOS by a clinical psychologist who had completed research certification in the measure. Following the consent, the order of the SADE and RDE visits were randomized.

In preparation for the dental visits, a social story was sent to parents to read to their children before the visits approximately 1–2 weeks before each visit. The purpose of the story was to help children accept electrode application on their fingers during the dental cleanings and expose them to the sensory adaptations. The social stories described the dental cleaning procedure, including application of electrodes. Visual, auditory, and tactile deep pressure environmental adaptations were included only for the story provided prior to the SADE visit. Parents were instructed in the administration of the social story at the time of the consent process.

Intervention

In the *Control condition* (RDE), the dental cleaning was administered in the standard manner in a small private dental room. Cleanings were completed by a dentist and included an oral examination, prophylaxis (dental cleaning), and fluoride application. In the *Experimental condition* (SADE), the same room and dental practitioners were utilized, but the SADE procedures were enacted to modify the sensory input the child experienced. The intervention was based on two theoretical frameworks: Multisensory Environments (Shapiro 2011) and Sensory Integration

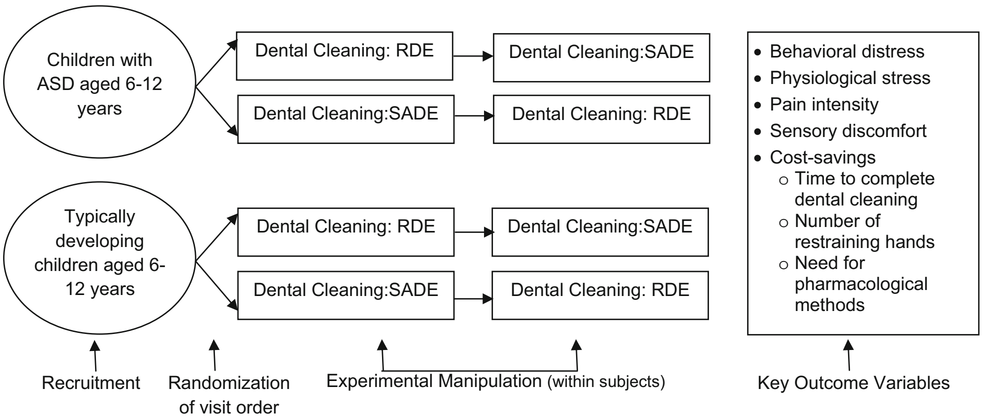

Fig. 1 Overview of study design. *RDE* Regular dental environment, *SADE* sensory adapted dental environment

Table 1 Descriptive characteristics of TD and ASD groups

	TD (n = 22) Mean (SD)	ASD (n = 22) Mean (SD)
Age (years)	8.3 (2.1)	8.2 (1.9)
Child and Adolescent Symptom Inventory–Anxiety Scale**	7.2 (5.9)	18.6 (9.1)
	N (%)	N (%)
*Gender**		
Male	10 (45.5)	18 (81.8)
Female	12 (54.5)	4 (18.2)
Race		
Caucasian	18 (81.8)	21 (95.5)
Not caucasian	4 (18.2)	1 (4.5)
Ethnicity		
Not hispanic, not latino	7 (31.8)	4 (18.2)
Hispanic, latino	15 (68.2)	18 (81.8)
Maternal education level		
High school, GED, or less	9 (40.9)	4 (18.2)
Vocational/associates/college courses	6 (27.3)	15 (68.2)
Bachelors degree or more	7 (31.8)	3 (13.6)
Paternal education level[a]		
High school, GED, or less	7 (33.3)	11 (52.4)
Vocational/associates/college courses	7 (33.3)	5 (23.8)
Bachelors degree or more	7 (33.3)	5 (23.8)
*VABS-II expressive language subtest of communication domain***		
Low ($\leq$2 percentile rank)	0 (0.0)	9 (40.9)
Moderately low (3–17 percentile rank)	3 (13.6)	11 (50.0)
Adequate (18–83 percentile rank)	14 (63.6)	2 (9.1)
Moderately high (84–94 percentile rank)	5 (22.7)	0 (0.0)
*Short Sensory Profile***		
Typical performance	14 (63.6)	0 (0.0)
Probable difference	7 (31.8)	1 (4.5)
Definite difference	1 (4.5)	21 (95.5)
*Children's Fear Survey Schedule—Dental Subscale***		
Non-clinical range	18 (81.8)	6 (27.3)
Borderline range	3 (13.6)	5 (22.7)
Clinical range	1 (4.5)	11 (50.0)

* $p \leq 0.05$; ** $p \leq 0.001$

[a] Missing data (n = 1 ASD group; n = 1 TD group); mother did not answer question

theory (Ayres 1972; Parham and Mailloux 2010), with the environmental modifications designed to minimize sensory-related discomforts and maximize relaxation. The following specific modifications to the dental room comprised the SADE intervention: (1) Visual: darkening curtains were placed on the windows and all direct overhead fluorescent lighting and the regular dental overhead lamp were turned off. One lamp was placed in the back corner of the room and projected into the curtain; this provided ambient light to enable camera recording. Slow moving visual color effects (Snoezelen) shone onto the ceiling in the child's visual field (swimming fish or bubbles, based on child preference). The dental practitioner wore a head-mounted lamp directed into the child's mouth, reducing bright lights shining in the child's eyes; (2) Auditory: rhythmic music was projected through portable speakers. The Music was Dan Gibson's Exploring Nature with Music (Gibson 1994); (3) Tactile (deep pressure): this stimulus consisted of a wrap designed to look like a butterfly, adapted from one developed by Shapiro et al. (2007), weighted with a regular pediatric dental X-ray vest. The wrap fit over the dental chair and was made of a washable material so it could be wiped down after each use. The wings of the butterfly wrapped around the child from shoulders to ankles and

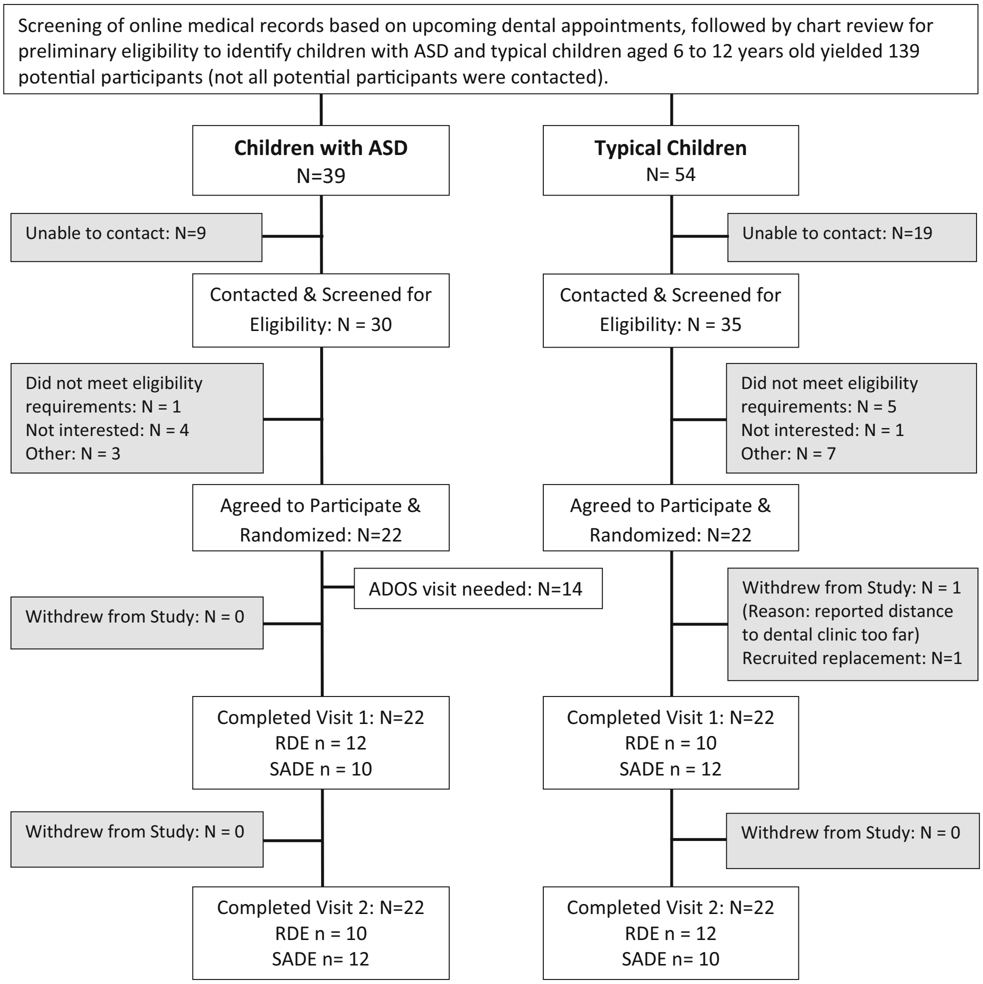

Fig. 2 Study flow chart

provided deep "hugging" pressure input designed to produce a calming effect (Edeslon et al. 1999; Grandin 2006). These wings were made of soft mesh-like breathable fabric so the participant would not become hot during the dental cleaning, were decorated with colorful felt circles, were fabricated in different sizes, and were detachable from the butterfly "body" allowing for different sized wings to be used with children. See Fig. 3. The main difference between the butterfly and a traditional papoose board is that the main body of the butterfly slips over the dental chair, thereby not requiring the child be strapped to a board such as that used for the papoose. Although the butterfly was not designed to provide protective stabilization, we recognize that it does so; therefore, we had a protocol in place such that children could request to keep open the butterfly wings or ask to open it during the cleaning. If this occurred, it was to be followed by verbal probing of the child and/or parent as to potential reasons for discomfort at the end of the cleaning. Two children required use of this protocol. The first, a 9.9 year old boy with ASD, upon entering the room immediately stated that he didn't want a butterfly hug. He sat on the butterfly-covered dental chair with the X-ray vest on his chest, and after the first half of the cleaning he requested "Can you use the wings?"; after the cleaning he stated that he liked the butterfly. His father reported that he had a "traumatic experience" with a papoose board at his last dental visit and attributed his son's initial apprehension of the butterfly hug to that incident. The second was a 12.8 year old typically developing female who, once the butterfly was closed, expressed some fear of "claustrophobia". She requested it be opened, but then decided that she wanted the butterfly hug with her arms outside the butterfly. When questioned at the end of the cleaning she stated that she was "more relaxed today than usual…I think it was everything [butterfly, lights, and music] put together" and that she "kind of" didn't want to leave the dentist because she felt like she was "tucked into bed".

Upon the completion of each cleaning, the child completed a pain intensity scale and a sensory discomfort measure; the dentist completed cooperation rating scales

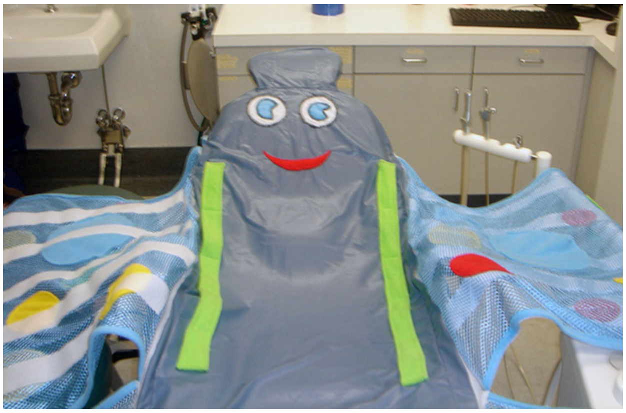

Fig. 3 "Butterfly" wrap placed on reclining dental chair with wings open

based on the child's exhibited behavior during the cleaning (see Instruments section).

In order to ensure intervention fidelity, dentists who treated participants underwent 2 h of training offered to the entire dental staff to understand the sensory characteristics of children with ASD and the principles underlying use of the multisensory environment in the SADE procedure. Training was provided by the lead author and by Shapiro, a consultant who developed the SADE protocol (Shapiro et al. 2009a, b). A fidelity checklist was developed and used in each session by a research assistant who also videotaped the session to monitor adherence to the SADE intervention, confirming that the lights, sounds, and butterfly wrap were used as per the protocol.

Child Descriptor Measures

Child descriptor measures were used to determine study eligibility and/or describe the participants.

Demographics

Demographic information regarding child's gender, age, ethnicity, race, and parental education level was obtained via the parent-report demographics questionnaire.

ASD Diagnosis

The *Autism Diagnostic Observation Schedule* (ADOS; Lord et al. 1999) is a semi-structured, standardized observational assessment of a child's social communication, reciprocal social interactions, and repetitive behaviors or interests. The ADOS was administered by a research-certified psychologist to confirm ASD diagnosis in children who had not been previously assessed with the ADOS.

Communication

The parent-report *Vineland Adaptive Behavior Scales II Expressive Language subtest* (VABS-II; Sparrow et al. 2005) was used to assess the child's communication competence. The Expressive Communication subscale discriminates among children with different levels of ASD severity and has served as a brief measure of expressive language in autism studies (Jones and Lord 2013).

Sensory Processing

The *Short Sensory Profile* (SSP; Dunn 1999) is the most frequently used assessment of sensory processing in children with ASD (Ben-Sasson et al. 2009b). It is a 38-item parent-report questionnaire standardized for children ages 3 through 10 years. Using a 5-point Likert scale, caregivers report how frequently their child responds to sensory input in daily life activities, with lower scores indicating greater sensory processing difficulties.

Anxiety

The *Child and Adolescent Symptom Inventory-Anxiety Scale* (CASI-Anx; Gadow and Sprafkin 1994) is a parent-report questionnaire assessing a range of anxiety symptoms, and has been modified and validated on a sample of children with autism aged 5–17 years, including children with and without intellectual disabilities (Sukhodolsky et al. 2008). Higher scores indicate greater anxiety.

The *Children's Fear Survey Schedule—Dental Subscale* (CFSS-DS; Cuthbert and Melamed 1982), is a 15 item self-report and/or parent-report scale for assessing dental anxiety, scored on a 5-point Likert scale from 1 (not afraid at all) to 5 (very afraid). Total scores range from 15 to 75, with scores above 32 indicating the presence of dental fear. This assessment has high reliability and validity, and normative data are available for children aged 4–14 years (Ten Berge et al. 2002a, b).

Outcome Measures

Our primary outcome measure was physiological stress and anxiety, measured by electrodermal activity (EDA). Secondary outcome measures included behavioral distress, pain intensity, sensory discomfort, and measures related to cost of dental procedure. Multiple measures were used to assess distress during dental procedures to account for the range of ages, developmental levels, and communication abilities of participants.

Physiological Stress and Anxiety

Electrodermal Activity (*EDA*) reflects the skin conductance of the palmar sweat glands controlled by the sympathetic nervous system. EDA was recorded continuously throughout the dental cleaning as well as for 3 min prior to the cleaning by placing pre-gelled electrodes on the index and middle fingertips of the child's non-dominant hand. In longer-lasting situations, such as a dental cleaning, measurement of tonic skin conductance level (SCL) and frequency of non-specific skin conductance responses (NS-SCRs) are the most useful electrodermal measures (Dawson et al. 2007); these measures exhibit significant test–retest reliability (temporal stability; r ranges from 0.40 to 0.85) when measured over a duration of a few days to a year or longer (Dawson et al. 2007; Schell et al. 2002; Schoen et al. 2008). It is well-documented that these tonic EDA readings increase in stressful or painful situations (Dawson et al. 2007).

Behavioral Distress

The *Children's Dental Behavior Rating Scale (CDBRS)* was developed and pilot tested by the authors prior to the initiation of this study (Peterson et al. 2013). Trained coders view video-tapes of dental cleanings and rate child behavior during the first 5 min of prophylaxis. Coded items included the presence or absence of three distress behaviors (mouth movement, head movement, forehead movement), and severity of two distress behaviors (whimper/cry/scream and verbal stall or delay). The raw score (0–45) is converted, via Rasch analysis, to a scale score of 1–100 for easier understanding, as higher scores indicate greater behavioral distress. Inter-rater reliability by two trained raters on a sample of 15 children with and without ASD (34 % of total sample) was $K = 0.97$, $p < 0.001$.

The Anxiety and Cooperation Scale (Veerkamp et al. 1995) is a dentist-report rating scale of patient behavior during treatment. The scale includes one question with a Likert-scale ranging from 0 (relaxed, smiling, demonstrates desired behavior, complies with demands) to 5 (out of control, loud crying, reverts to primitive flight responses, physical restraint required) such that lower scores indicate greater cooperation and relaxation while higher scores denote greater behavioral distress. The Scale has been shown to assess changes in children's anxiety, fear, and cooperation and has established reliability and validity (Schriks and van Amerongen 2003; Versloot et al. 2008).

The *Frankl Scale* (Frankl et al. 1962) is a dentist-report measure utilized by researchers and clinicians to score behavior in the dental environment. It includes a one-item Likert Scale ranging from 1 (definitely negative) to 2 (negative) to 3 (positive) to 4 (definitely positive) and has high inter-rater reliability and moderate validity (Aartman et al. 1996).

Pain Intensity

The *Faces Pain Scale-Revised (FPS-R*; Hicks et al. 2001) was used to assess the intensity of pain experienced during the dental cleaning. This child-report scale consists of drawings of six faces that express increasing distress, with the child asked to point to the face that best fits his or her level of pain from "*no pain*" to "*very much pain.*" The six levels of pain reflect a rating scale that ranges from 0 (no pain) to 10 (very much pain; increments of 2). Validity of the measure is supported by a high positive correlation with a visual analogue scale in children aged 5–12 years (Hicks et al. 2001; Tomlinson et al. 2010).

Sensory Discomfort

The *Dental Sensory Sensitivity Scale* is a child-report survey developed for this study assessing the extent of "bother" of the different sensory stimuli exposed to during a dental cleaning (e.g., lights, sounds, smell, taste, vestibular input, total "bother"). There are six items, with each scored on a three-point Likert Scale (no bother, a little bother, a lot of bother) and higher scores indicating greater extent of "bother".

Cost Savings

To calculate cost-savings, *Duration of dental cleaning* was calculated from video recordings of the treatment sessions. Timing began when the dentist started the dental examination and ended when the fluoride application was completed. Time for EDA electrode application and rest periods were subtracted.

As another measure of cost, the number of people required to restrain the child during the cleaning was documented using a measure that we called *Number of hands*. This was the maximum number of hands at any one time needed to hold or restrain the child. *Need for pharmacological methods* reflected whether the cleaning could be completed or whether a return visit under sedation, anesthesia, or any other pharmacological method was required.

Data Analysis

For electrodermal activity data, the number of non-specific skin conductance responses (NS-SCRs) were totaled for each participant and converted to a rate of fluctuations per minute; NS-SCRs were counted only when the amplitude was greater than or equal to 0.05 µS, as suggested by

Dawson et al. (2007). As is common practice, tonic skin conductance level was transformed prior to analysis to reduce the skew and kurtosis of the data with a logarithmic transformation (Dawson et al. 2007). Both SCL and NS-SCRs were computer scored off-line using the BIOPAC program Acq*Knowledge* and hand-checked to ensure no skin conductance responses were missed or incorrectly marked; 25 % of the hand-coded data were double coded to ensure that the identification of NS-SCRs was reliable, with 96 % agreement (calculated as the number of matching NS-SCRs divided by total number of NS-SCRs coded by the researchers).

Child and family characteristics of the ASD and TD groups were compared using *t* tests for continuous variables and Chi square for categorical variables. Intent to treat analyses were performed using repeated measures ANCOVA to test the effect of two factors: dental environment (within) and autism diagnosis (between) and the interaction of these two variables. As this is a pilot study, and thus statistical significance may not prove a good indicator of efficacy, effect sizes (Cohen's d) were computed for effect of dental environment (RDE vs. SADE) within each group (TD vs. ASD) to indicate the strength of the treatment effect. The randomized order of visits was included as an a priori covariate for comparisons of dental environments. Analyses were performed with SPSS (v.21).

Results

Study results indicate that utilizing a sensory adapted dental environment during dental cleanings for children is indeed feasible, with 100 % adherence to the aforementioned fidelity checklist.

Response to Intervention: Physiological Measures

As shown in Table 2, there were decreases in EDA in the SADE condition compared to the RDE for both ASD and TD groups. Repeated measures ANCOVA models found a statistically significant effect of environment for SCL exam + prophylaxis + fluoride and exam + prophylaxis (p's $= 0.01$), and NS-SCR exam + prophylaxis ($p = 0.05$). There were statistically significant group differences between ASD and TD for all EDA measures, SCL exam + prophylaxis + fluoride ($p = 0.05$) and exam + prophylaxis ($p = 0.06$), and for the NS-SCR measures (p's < 0.01; See Fig. 4). There were no statistically significant environment by group interactions, which is not surprising given the pilot nature of this study. Effect sizes for our primary outcomes, measured by skin electrodermal activity (EDA), were moderate in the TD group (d's = 0.30–0.46) and moderate to large in the ASD group (d's = 0.27–0.65).

Response to Intervention: Behavioral Measures

As anticipated, ANCOVA models showed statistically significant group effects for all behavioral measures (all p's < 0.03). Environment effects on behavioral measures (Table 2) were not statistically significant, though all were in the hypothesized direction with primarily small effects (d's < 0.3). The largest impact of environment was seen in the CDBRS, which entails observer coding of child's distress behaviors; the effect size of the SADE versus RDE on this measure of behavioral distress was 0.29 for the TD group and 0.23 for children with ASD.

There were noticeable differences in effect size between groups in the within environment change in the dentist-report measures. The Anxiety and Cooperation scale showed an effect size of 0.13 in ASD children and 0.06 in TD. When ratings were dichotomized into *Relaxed/Cooperative* (0–2) vs. *Stressed* (3–5), the TD children were scored as very relaxed in both settings (91 % in RDE and 96 % in SADE), while there was an increase in being rated as relaxed and cooperative in the SADE condition for the ASD group (46 % RDE to 59 % SADE). With this measure dichotomized, the environment effect was notable ($p = 0.08$). The dentist-report Frankl Scale also showed effects in the ASD group between environments (d = 0.21); effect size for environment were very small in the TD and small in the ASD group. As with the Anxiety and Cooperation Scale, when stratified into *Negative* (1–2) and *Positive* (3–4) categories, most of the TD group exhibited positive behavior in both environments (91 and 95 % for RDE and SADE, respectively), while there was an increase in positive behavior rating in the ASD group from 54 % in the RDE to 64 % in the SADE environment.

Response to Intervention: Child-Report Measures

The child-report measures of pain intensity (FPS-R) and sensory discomfort in the dental environment (DSSS) were significantly improved in both ASD and TD groups in the SADE environment (p's $= 0.05$ and 0.09, respectively). The effect sizes were in the moderate to large range for the ASD group, and in the small to moderate range for the TD group. There were not statistically significant group differences in this measure, and in the SADE condition, the means were almost identical between TD and ASD children. However, these results must be interpreted with caution as all TD children reported on both measures in both environments but only about half the children with ASD were able to complete the self-report ratings (n = 13 in RDE, n = 10 in SADE); therefore, this finding represents only a partial sample of our ASD group.

Table 2 Study outcomes by group and condition

	TD (n = 22)			ASD (n = 22)		
	RDE<br>M ± SD	SADE<br>M ± SD	Effect size	RDE<br>M ± SD	SADE<br>M ± SD	Effect size
Physiological outcomes						
Skin conductance level						
Exam + prophy + fluoride	4.2 ± 3.9	2.7 ± 1.9	0.42	5.6 ± 3.9	3.6 ± 2.7	0.65
Exam + prophy	4.3 ± 3.8	2.7 ± 1.8	0.46	5.6 ± 3.8	3.6 ± 2.5	0.62
Non-specific skin conductance responses						
Exam + prophy + fluoride	3.0 ± 2.5	2.2 ± 2.4	0.30	5.8 ± 3.3	4.6 ± 5.3	0.27
Exam + prophy	3.3 ± 2.5	2.3 ± 2.3	0.40	6.1 ± 3.5	4.4 ± 3.8	0.46
Behavioral outcomes						
CDBRS scaled	34.7 ± 12.5	30.8 ± 14.6	0.29	47.3 ± 8.6	44.9 ± 11.8	0.23
A & C scale	0.5 ± 1.1	0.4 ± 1.1	0.06	2.1 ± 1.6	1.8 ± 2.0	0.13
Frankl scale	3.6 ± 0.7	3.64 ± 0.7	0.04	2.5 ± 1.0	2.7 ± 1.2	0.21
Child-report outcomes						
Pain intensity	0.3 ± 0.7	0.1 ± 0.3	0.49	0.6 ± 1.2	0.1 ± 0.3	0.62
DSSS	2.6 ± 1.5	2.2 ± 2.5	0.18	3.9 ± 2.4	2.3 ± 2.3	0.69
Measures of cost						
Duration of cleaning (min)	30.7 ± 6.0	38.1 ± 12.9	0.78	32.1 ± 7.3	37.4 ± 5.9	0.80
Number of hands	–	–	–	3 ± 3	1.9 ± 2.2	0.42

RDE Regular dental environment, *SADE* sensory adapted dental environment; Effect size is calculated for the dental environments within each group, adjusted for order of visit, and are Cohen's D. For SCL, log values were used for statistics, though averages of raw values by minute are presented here. Number of hands was not applicable to the TD group, as only 1 child required restraining hands during treatment

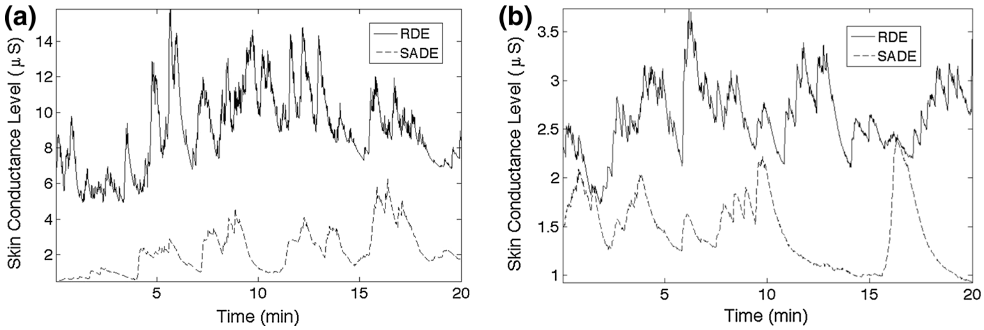

Fig. 4 Skin conductance level of the first 20 min of dental cleanings in the regular and sensory adapted dental environments in **a** one participant with ASD, and **b** one TD participant. *RDE* Regular dental environment, *SADE* sensory adapted dental environment. Please note that the y-axis scale is different for the child with ASD (intervals of 2μS) and the TD child (intervals of 0.5 μS)

Cost Savings

Three measures were used to examine possible cost savings. First was the length of cleaning. Duration of cleaning was significantly longer in the SADE condition (mean difference 5–7 min for both groups), opposite of the hypothesized direction. However, the number of hands required to restrain the child during cleaning was significantly reduced in the SADE condition. As only one TD child required restraint, this represents the ASD group (effect size 0.42). Third was requiring pharmacological methods to complete the dental cleaning. None of the study participants required these interventions during the study, although a number of children in the ASD group had a history of requiring general anesthesia.

Discussion

The SADE intervention showed promising results. With respect to our first hypothesis, our findings supported that children with ASD exhibit greater behavioral and physiological distress during dental cleanings in both the RDE and SADE, compared to TD children. This is consistent with numerous reports from parents who describe the behavioral challenges of bringing their child with ASD to the dentist (Stein et al. 2012a, b) as well as supported by research indicating differences in physiological and behavioral distress in children with ASD as compared to TD children undergoing routine dental cleanings at the dentist (Stein et al. 2014).

Our second hypothesis was partially supported. We found that children with ASD and, to a lesser extent, TD children exhibited less physiological distress during the SADE cleaning compared to the RDE; the majority of the behavioral distress measures also indicated a decrease in distress in the SADE compared to the RDE.

It is important to note that this research was designed as a feasibility and pilot study and therefore not powered to detect differences between the two dental environments, with small sample sizes limiting any definitive conclusions about the efficacy of the sensory adapted dental environment. However, our findings were in the expected direction, enabling us to examine trends and estimate the sample size required for an adequately powered large-scale randomized controlled trial. Additionally, a strength of this study was the ability to utilize strategies with participants and their families to diminish attrition and drop-out; as seen in Fig. 2, 98 % of consented participants were retained.

Our third hypothesis was that the SADE environment would require a shorter duration for dental cleaning and fewer personnel, thus reducing cost; the findings produced mixed results. Three factors affect the cost of a dental cleaning: the length of time to complete the cleaning, the number of personnel required to complete the cleaning, and the need for pharmacological methods. In this study, the SADE did not result in a shortened time to complete the dental cleaning. In fact, we found the opposite, with dental cleanings in the SADE condition taking an average of 5–7 min longer. However, time may not be the most appropriate variable to measure; as highlighted by the dentists participating in the study, when a child was more cooperative the dentist may have been able to dedicate more time to clean the child's teeth. Thus, although the cleaning time is longer, the cleaning may be more effective. This was not examined in the present study and should be investigated in future research. The second factor contributing to cost effectiveness is the number of personnel required to successfully complete a dental cleaning. In the RDE, significantly more people were needed to restrain children with ASD in order to complete the cleaning compared to the SADE, reflecting an increase in cost in the RDE. In this study, all children were able to complete the cleaning and did not require anesthesia (the third cost factor), even though some of the children had required anesthesia for previous cleanings. It is possible that use of the social story, which was used for both conditions, may have also prepared the child for the dental visit.

Feedback from the participating dental practitioners indicated that it is feasible to incorporate the SADE intervention into regular dental practice. The equipment costs were minimal, no permanent renovations were required to the dental clinic, and the equipment was portable and easy to set-up and remove. The development of a modified dental environment may therefore be a promising approach to enable greater numbers of pediatric dentists as well as general dentists trained in advanced behavioral guidance techniques to reduce common dental behavior management problems that occur when treating children with ASD. Because general dentists indicate that behavior problems are the greatest barrier to their willingness to treat children with disabilities (Casamassimo et al. 2004), decreasing children's distress behaviors may increase dentists' willingness to treat children with ASD. Additionally, treatment may become safer for the child if there is a reduction in the use of general anesthesia, which is more frequently used when children are uncooperative during dental treatment (Loo et al. 2009). Moreover, the need for restraint (i.e., protective stabilization; AAPD, 2010-11) may also be reduced. Research currently indicates that restraint is utilized for children with ASD 18–33 % of the time, significantly more than with typically developing children (3 %) and many parents object to this restraint (Marshall et al. 2008; Stein et al. 2012a). In the present study, parents reported an increase in acceptance for the "butterfly" compared to previous use of restraint with a papoose board for their child. Moreover, the butterfly included the added benefits of deep pressure tactile stimulation, which has been identified by individuals with ASD as having a calming effect (Edeslon et al. 1999; Grandin 2006). Finally, as ease of cleaning and experience improves, parents may be more likely to bring their child to the dentist for routine oral care, resulting in enhanced oral health.

There are three primary areas to investigate in future research of the SADE. First, it will be important to determine if the positive results of the adapted environment are due to the treatment package as a whole (e.g., visual, auditory, and tactile stimuli adaptations), or if one or two components are sufficient to obtain positive results. Second, the idea of combining sensory and behavioral intervention components is also worthy of future study in order to determine if the

effects of a combined intervention are more beneficial than either treatment individually. Lastly, it is essential to examine whether the SADE intervention may also be helpful for children with other disabilities as well as for typically developing children with dental fear or sensory sensitivities, as the intervention provides an environment that is less sensory aversive and reduces physiological stress and anxiety. Research has indicated that 9–26 % of typically developing children demonstrate significant dental fear and anxiety (Caprioglio et al. 2009; Gustafsson et al. 2010; Klingberg 2008) and between 5-33 % of typical children experience difficulty with sensory processing (Ahn et al. 2004; Ben-Sasson et al. 2009a; Leekam et al. 2007; Tomchek and Dunn 2007). In this study, 18 % of parents of TD children reported that their child experienced dental fear and anxiety (CFSS-DS scores in the borderline anxiety range), and 36 % of TD children had differences in sensory processing (SSP scores in the probable difference range). In this regard, it is noteworthy that in Shapiro et al.'s (2007, 2009a, b) investigation, the SADE had statistically reliable positive effects (reductions in the duration of anxious behaviors and physiological arousal) for typically developing children, although those effects were smaller than those found for children with developmental disabilities; results in the present study followed a similar pattern.

Limitations

We have identified four limitations to this study. The *first limitation* pertains to recruitment in two ways: ASD severity and gender distribution. (a) As we recruited all children with a diagnosis of ASD regardless of severity, we expected to have a range of functional levels. However, as noted in Table 1, 91 % of the participants in the ASD group had expressive communication skills significantly below average, indicating a lower functioning sample. We expect that this may be indicative of the children with ASD who receive their dental care in a hospital setting. Therefore, in order to generalize to the broader ASD population, we will need to include children with high functioning autism in a future study, allowing us to examine if cognitive/language level moderates the effect of the intervention. (b) This study consisted of a consecutive sample of consenting patients and parents; therefore, the gender distribution of the TD group was approximately 1:1 while the male to female distribution in the ASD group was 4.5:1. Although consistent with national statistics (CDC 2014), this gender difference may have impacted the study results. The *second limitation* regards outcome measures, in three ways. (a) Approximately half of the children in the ASD group were unable to complete self-report measures. Previous research does indicate that youth and adolescents with ASD can successfully self-report pain utilizing faces pain scales (Moore 2015). However, due to the introspection required to self-report pain this task may be difficult in children with ASD; therefore, it is suggested that finding should be supplemented by other measures (Moore 2015). In anticipation of this possibility, we included measures from the dentist, researcher, and child perspectives as well as the collection of physiological data. Inclusion of children who are higher functioning in future studies will increase child reporting. (b) Some of the study outcome measures were not blinded to intervention. This type of issue is endemic to all intervention versus control comparisons that involve dyadic therapeutic-client interaction, but does not preclude the ability to perform controlled trials. Based on the obvious environmental manipulations or lack thereof of this intervention, it was impossible to blind some of the outcome measures, such as the dentist-report of child behavior. However, the video-coding of behavior utilizing the CDBRS was blinded to group; we were unable to blind to condition because the SADE was identifiable in videos based on the lighting and music present. (c) This study did not include a measure of thoroughness of dental cleaning; in future studies, this should be investigated as a way to determine if the longer duration of dental cleaning in the SADE was, in fact, indicative of a more thorough dental cleaning as suggested by the dental team. The *third limitation* notes that providing children with a social story about the dental cleaning ahead of time may have led to unanticipated decreases in physiological and behavioral distress in the children. However, the social story was integral in familiarizing the children with the EDA electrodes and gaining their acceptance of the electrode application and use throughout the dental cleaning. Lastly, the *fourth limitation* focuses on our small sample size, which precluded us from examining the specific effects of dental anxiety, sensory over-responsivity, and other mediating and moderating factors such as cognitive/language ability on the efficacy of the sensory adapted dental environment. A larger sample in future studies will enable us to examine these factors and determine which children will best benefit from the SADE intervention.

Conclusion

Use of the sensory adapted dental environment shows utility and positive treatment effect sizes. The use of this type of dental environment has the potential to not only improve dental care for children with ASD, but for children with other disabilities, and typically developing children with dental anxiety and/or sensory processing difficulties. Further research is needed to examine which factors may moderate treatment efficacy.

Acknowledgments This study was funded by the National Institute of Dental and Craniofacial Research (1R34DE022263-01; ClinicalTrials.gov identifier: NCT02077985) and by a seed grant from the Ostrow School of Dentistry; support was also provided by the Eunice Kennedy Shriver Institute of Child Health and Human Development of the National Institutes of Health (T32HD064578). We would like to thank: the University of Southern California Division of Occupational Science and Occupational Therapy for their continued support; Michele Shapiro, PhD, OTR and Anat Baniel, DDS from Beit Issie Shapiro, Ra'nana, Israel for providing consultation throughout this project; Trudy Mallinson, PhD, OTR/L for her assistance with Rasch Analysis in developing the CDBRS coding scale; Irina Zamora, PsyD for assistance in confirming diagnosis of children with ASD; Elyse Peterson, OTD, OTR/L and Lauren St. Hilaire, OTD, OTR/L for their assistance in psychometric testing of the CDBRS, data collection, and inter-rater reliability assessments of measures; and Daniella Floríndez for her unmatched translating and interpreting skills.

Conflict of interest The authors declare that there are no conflicts of interest regarding the publication of this paper.

References

Aartman, I. H. A., van Everdingen, T., Hoogstraten, J., & Schuurs, A. H. B. (1996). Appraisal of behavioral measurement techniques for assessing dental anxiety and fear in children: A review. *Journal of Psychopathology and Behavioral Assessment, 18*, 153–171.

Ahn, R. R., Miller, L. J., Milberger, S., & McIntosh, D. N. (2004). Prevalence of parents' perceptions of sensory processing disorders among kindergarten children. *American Journal of Occupational Therapy, 58*, 287–293.

American Psychiatric Association. (2013). *Diagnostic and statistical manual of mental disorders* (5th ed.). Arlington, VA: American Psychiatric Publishing.

Ayres, A. J. (1972). *Sensory integration and learning disorders*. Los Angeles: Western Psychological Services.

Baker, A. E. Z., Lane, A., Angley, M. T., & Young, R. I. (2008). The relationship between sensory processing patterns and behavioural responsiveness in autistic disorder: A pilot study. *Journal of Autism and Developmental Disorders, 38*, 867–875.

Baranek, G. T., David, F. J., Poe, M. D., Stone, W. L., & Watson, L. R. (2006). Sensory experiences questionnaire: Discriminating sensory features in young children with autism, developmental delays, and typical development. *Journal of Child Psychology and Psychiatry, 47*, 591–601.

Ben-Sasson, A., Carter, A. S., & Briggs-Gowan, M. J. (2009a). Sensory over-responsivity in elementary school: Prevalence and social-emotional correlates. *Journal of Abnormal Child Psychology, 37*, 705–716.

Ben-Sasson, A., Hen, L., Fluss, R., Cermak, S. A., Engel-Yeger, B., & Gal, E. (2009b). A meta-analysis of sensory modulation symptoms in individuals with autism spectrum disorders. *Journal of Autism and Developmental Disorders, 39*, 1–11.

Brickhouse, T. H., Farrington, F. H., Best, A. M., & Ellsworth, C. W. (2009). Barriers to dental care for children in Virginia with autism spectrum disorders. *Journal of Dentistry for Children, 76*, 188–193.

Caprioglio, A., Mariani, L., & Tettamanti, L. (2009). A pilot study about emotional experiences by using CFSS-DS in young patients. *European Journal of Paediatric Dentistry, 10*, 121–124.

Casamassimo, P. S., Seale, N. S., & Ruehs, K. (2004). General dentists' perceptions of educational and treatment issues affecting access to care for children with special health care needs. *Journal of Dental Education, 68*, 23–28.

Centers for Disease Control and Prevention (2014). Autism spectrum disorders: Data and statistics. http://www.cdc.gov/ncbddd/autism/data.html

Cuthbert, M. I., & Melamed, B. G. (1982). A screening device: Children at risk for dental fears and management problems. *Journal of Dentistry for Children, 49*, 432–436.

Dao, L. P., Zwetchkenbaum, S., & Inglehart, M. R. (2005). General dentists and special needs patients: Does dental education matter? *Journal of Dental Education, 69*, 1107–1115.

Dawson, M. E., Schell, A. M., & Filion, D. L. (2007). The electrodermal system. In J. T. Cacioppo, L. G. Tassinary, & G. G. Bernston (Eds.), *Handbook of psychophysiology* (3rd ed., pp. 159–181). Cambridge: Cambridge University Press.

Dunn, W. (1999). *Sensory profile: User manual*. USA: The Psychological Corporation.

Edelson, S. M., Edelson, M. G., Kerr, D. C. R., & Grandin, T. (1999). Behavioral and physiological effects of deep pressure on children with autism: A pilot study evaluating the efficacy of Grandin's Hug Machine. *American Journal of Occupational Therapy, 53*, 145–152.

Frankl, S. N., Shiere, F. R., & Fogels, H. R. (1962). Should the parent remain with the child in the dental operatory. *Journal of Dentistry for Children, 29*, 150–163.

Gadow, K. D., & Sprafkin, J. (1994). *Child symptom inventory-4*. Stony Brook: Checkmate Plus.

Geschwind, D. H. (2009). Advances in autism. *Annual Review of Medicine, 60*, 367–380.

Gibson, D. (1994). Solitudes: Exploring nature with music. http://www.allmusic.com/album/the-classics-2-exploring-nature-with-music-mw0000916892

Grandin, T. (2006). *Thinking in pictures: My life with autism*. New York: Vintage Books.

Gustafsson, A., Arnrup, K., Broberg, A. G., Bodin, L., & Berggren, U. (2010). Child dental fear as measured with the Dental Subscale of the Children's Fear Survey Schedule: The impact of referral status and type of informant (child versus parent). *Community Dentistry and Oral Epidemiology, 38*, 256–266.

Hicks, C. L., von Baeyer, C. L., Spafford, P. A., van Korlaar, I., & Goodenough, B. (2001). The Faces Pain Scale-Revised: Toward a common metric in pediatric pain measurement. *Pain, 93*, 173–183.

Klingberg, G. (2008). Dental anxiety and behavior management problems in paediatric dentistry: A review of background factors and diagnostics. *European Archives of Paediatric Dentistry, 9*(Suppl 1), 11–15.

Kopycka-Kedzierawski, D. T., & Auinger, P. (2008). Dental needs and status of autistic children: Results from the national survey of children's health. *Pediatric Dentistry, 30*, 54–58.

Leekam, S. R., Nieto, C., Libby, S. J., Wing, L., & Gould, J. (2007). Describing the sensory abnormalities of children and adults with autism. *Journal of Autism and Developmental Disorders, 37*, 894–910.

Loo, C. Y., Graham, R. M., & Hughes, C. V. (2009). Behaviour guidance in dental treatment of patients with autism spectrum disorder. *International Journal of Paediatric Dentistry, 19*, 390–398. doi:10.1111/j.1365-263X.2009.01011.x.

Lord, C., Rutter, M., DiLavore, P., & Risi, S. (1999). *Autism Diagnostic Observation Schedule Manual*. Los Angeles: Western Psychological Services.

Marshall, J., Sheller, B., Mancl, L., & Williams, B. J. (2008). Parental attitudes regarding behavior guidance of dental patients with autism. *Pediatric Dentistry, 30*, 400–407.

Moore, D. J. (2015). Acute pain experience in individuals with autism spectrum disorders: A review. *Autism, 19*, 387–399.

Nelson, L. P., Getzin, A., Graham, D., Zhou, J., Wagle, E. M., McQuiston, J., & Huntington, N. L. (2011). Unmet dental needs and barriers to care for children with significant special health care needs. *Pediatric Dentistry, 33*, 29–36.

Parham, D., & Mailloux, Z. (2010). Sensory integration. In J. Case-Smith & J. O'Brien (Eds.), *Occupational therapy for children* (6th ed.). Maryland Heights, MO: Mosby.

Peterson, E., Stein, L. I., Polido, J. C., Mallinson, T., & Cermak, S. A. (2013). Developing an instrument to assess children's distress behaviors in the dental environment [Abstract]. *The Explorer: Journal of USC Student Research, 5*, 53.

Schell, A. M., Dawson, M. E., Nuechterlein, K. H., Subotnik, K. L., & Ventura, J. (2002). The temporal stability of electrodermal variables over a one-year period in patients with recent-onset schizophrenia and in normal subjects. *Psychophysiology, 39*, 124–132. doi:10.1111/1469-8986.3920124.

Schoen, S. A., Miller, L. J., Brett-Green, B., & Hepburn, S. L. (2008). Psychophysiology of children with autism spectrum disorder. *Research in Autism Spectrum Disorders, 2*, 417–429. doi:10.1016/j.rasd.2007.09.002.

Schriks, M. C. M., & van Amerongen, W. E. (2003). A traumatic perspectives of ART: Psychological and physiological aspects of treatment with and without rotary instruments. *Community Dental Oral Epidemiology, 31*, 15–20.

Shapiro, M. (2001). *Beit Issie Shapiro's approach to multi-sensory environments (Snoezelen)*. Ra'anana, Israel: Rotem Publishing.

Shapiro, M., Melmed, R. N., Sgan-Cohen, H. D., Eli, I., & Parush, S. (2007). Behavioural and physiological effect of dental environment sensory adaptation on children's dental anxiety. *European Journal of Oral Science, 115*, 479–483. doi:10.1111/j.1600-0722.2007.00490.x.

Shapiro, M., Melmed, R. N., Sgan-Cohen, H. D., & Parush, S. (2009a). Effect of sensory adaptation on anxiety of children with developmental disabilities: A new approach. *Pediatric Dentistry, 31*, 222–228.

Shapiro, M., Sgan-Cohen, H. D., Parush, S., & Melmed, R. N. (2009b). Influence of adapted environment on the anxiety of medically treated children with developmental disability. *Journal of Pediatrics, 154*, 546–550.

Sparrow, S. S., Cicchetti, D. V., & Balla, D. A. (2005). *Vineland Adaptive Behavior Scales* (2nd ed.). Circle Pines, MN: American Guidance Service.

Stein, L. I., Lane, C. J., Williams, M. E., Dawson, M. E., Polido, J. C., & Cermak, S. A. (2014). Physiological and behavioral stress and anxiety in children with autism spectrum disorders during routine oral care. *BioMed Research International,* 2014, Article ID 694876. doi:10.1155/2014/694876.

Stein, L. I., Polido, J. C., & Cermak, S. A. (2012a). Oral care and sensory concerns in autism. *American Journal of Occupational Therapy, 66*, e73–e76. doi:10.5014/ajot.2012.004085.

Stein, L. I., Polido, J., & Cermak, S. (2013). Oral care and sensory over-responsivity in children with autism spectrum disorder. *Pediatric Dentistry, 35*, 230–235.

Stein, L. I., Polido, J. C., Mailloux, Z., Coleman, G. G., & Cermak, S. A. (2011). Oral care and sensory sensitivities in children with autism spectrum disorders. *Special Care in Dentistry, 31*, 102–110. doi:10.1111/j.1754-4505.2011.00187.x.

Stein, L. I., Polido, J. C., Najera, S. O., & Cermak, S. A. (2012b). Oral care experiences and challenges in children with autism spectrum disorder. *Pediatric Dentistry, 34*, 387–391. doi:10.5014/ajot.2012.004085.

Sukhodolsky, D. G., Scahill, L., Gadow, K. D., Arnold, L. E., Aman, M. G., McDougle, C. J., & Vitiello, B. (2008). Parent-rated anxiety symptoms in children with pervasive developmental disorders: Frequency and association with core autism symptoms and cognitive functioning. *Journal of Abnormal Child Psychology, 36*, 117–128. doi:10.1007/s10802-007-9165-9.

Ten Berge, M., Veerkamp, J. S. J., Hoogstraten, J., & Prins, P. J. M. (2002a). Childhood dental fear in the Netherlands: Prevalence and normative data. *Community Dentistry and Oral Epidemiology, 30*, 101–107.

Ten Berge, M., Veerkamp, J. S. J., Hoogstraten, J., & Prins, P. J. M. (2002b). The Dental Subscale of the Children's Fear Survey Schedule: Predictive value and clinical usefulness. *Journal of Psychopathology and Behavioral Assessment, 24*, 115–118.

Tomchek, S. D., & Dunn, W. (2007). Sensory processing in children with and without autism: A comparative study using the short sensory profile. *American Journal of Occupational Therapy, 61*, 190–200.

Tomlinson, D., vonBaeyer, C. L., Stinson, J. N., & Sung, L. (2010). A systematic review of Faces Scales for the self-report of pain intensity in children. *Pediatrics, 126*, e1168. doi:10.1542/peds.2010-1609.

U.S. Department of Health & Human Services (2010). *Healthy People 2020, Oral Health: Overview, objectives, and interventions and resources*. http://www.healthypeople.gov/2020/topicsobjectives2020/overview.aspx?topicid=32

Veerkamp, J. S. J., Gruythuysen, R. J. M., van Amerongen, W. E., Hoogstraten, J., & Weerheijm, K. I. (1995). Dentist's ratings of child dental-patients' anxiety. *Community Dentistry and Oral Epidemiology, 23*, 356–359.

Versloot, J., Veerkamp, J. S. J., & Hoogstraten, J. (2008). Dental anxiety and psychological functioning in children: Its relationship with behaviour during treatment. *European Archives of Paediatric Dentistry, 9*(Suppl. 1), 36–40. doi:10.1007/BF03262654.

Weil, T. N., & Inglehart, M. R. (2010). Dental education and dentists' attitudes and behavior concerning patients with autism. *Journal of Dental Education, 74*, 1294–1307.
