# Supplementary material for: Management strategies of dental anxiety and uncooperative behaviors in children with Autism spectrum disorder
Source: BMC Pediatr. 2023 Dec 4;23:612. doi: 10.1186/s12887-023-04439-7 (PMC10694959; doi:10.1186/s12887-023-04439-7)
Supplement: Supplementary file 4 — Additional file 4. Training children with autism spectrum disorder to undergo oral assessment using a digital iPad ® application [file 12887_2023_4439_MOESM4_ESM.pdf]

European Archives of Paediatric Dentistry
https://doi.org/10.1007/s40368-018-0398-9

ORIGINAL SCIENTIFIC ARTICLE

Training children with autism spectrum disorder to undergo oral assessment using a digital iPad® application

G. Lefer[1] · A. Rouches[2] · P. Bourdon[1,3] · S. Lopez Cazaux[1,2,4]

Received: 7 March 2017 / Accepted: 1 December 2018
© European Academy of Paediatric Dentistry 2018

Abstract

Aim To present a training programme for teaching children and adolescents with autism spectrum disorder (ASD), to be compliant with a dental examination.

Methods Fifty-two children and adolescents with ASD (age range 3–19 years) with a parent-signed consent form were enrolled. Dental examinations were performed once a month in education centres by a paediatric dentist using a visual activity schedule on an iPad® that was created with a digital application, çATED. Achievement and anxiety were evaluated using scales and grids every 2 months for 8 months.

Results Showed an improvement in oral assessment; the children became compliant and less anxious. The percentage of individuals who underwent the entire dental exam process increased over time; it was 25% at the beginning of the study and 65.4% after 8 months. Only 7.7% of the sample was not anxious at the beginning, while 59.6% of the sample was not anxious after 8 months. Wilcoxon analysis also showed significant improvement in the studied variables.

Conclusions Training children and adolescents with ASD to undergo dental examination was efficient. The use of the iPad® is attractive and easy for practitioners and people with ASD.

Keywords Autism · Oral health · Dental examination · iPad® · Training program

Introduction

Autism spectrum disorder (ASD) includes a group of developmental disabilities characterised by impaired social interaction and communication, the presence of repetitive and stereotyped behaviours and restricted interests (American Psychiatric Association 2013). These features impact the oral health of these individuals, and a high risk of dental caries, poorer periodontal status, bruxism and oral self-injury are often described (Loo et al. 2009; Marshall et al. 2010; Jaber 2011; Gandhi and Klein 2014).

Children with ASD often poorly cooperate with medical procedures, particularly those considered invasive, such as dental care (Cagetti et al. 2015). Recent studies have expanded our knowledge of the difficulties with home oral care and dental visits (Lai et al. 2012; Stein et al. 2012; Weil and Ingehart 2012; Barry et al. 2014; Lewis et al. 2015; Nelson et al. 2015). Children with ASD are prone to agitation, self-injury and emotional dysregulation; they can also present with hypersensitivity to sensory input (Brickhouse et al. 2009; Stein et al. 2013; Lewis et al. 2015). Therefore, treating children with ASD is challenging for the dental community.

In paediatric dentistry, communication and demonstration of dental procedures are critical to behaviour management (American Academy of Pediatric Dentistry 2016). However, these techniques have mostly failed with ASD patients. The use of adapted and specific strategies is needed to allow individuals with ASD to overcome the barriers of dental care.

Different tools and techniques can be considered because they are evidence-based practices. Early intensive

✉ S. Lopez Cazaux
serena.lopez-cazaux@univ-nantes.fr

1 Centre de Recherche en Education de Nantes, EA2661, Chemin de la Censive du Tertre, BP 81227, 44312 Nantes Cedex 3, France

2 Faculté de Chirurgie Dentaire, 1 Place Alexis Ricordeau, BP, 44042 Nantes Cedex 1, France

3 Ecole Supérieure du Professorat et de l'Education, 4 Chemin de Launay Violette, 44300 Nantes, France

4 Centre Hospitalo-Universitaire de Nantes, 1 Place Alexis-Ricordeau, 44093 Nantes Cedex 1, France

behavioural interventions are well established approaches to treat children with ASD and to teach them new skills (Reichow et al. 2012); repetitive and progressive approaches using positive reinforcement can be used, and some authors have discussed in vivo desensitisation (Conyers et al. 2004; Cuvo et al. 2010; Kemp 2015). These behavioural approaches are effective in the dental context (Loo et al. 2009; Cuvo et al. 2010; Hernandez and Ikkanda 2011; Delli et al. 2013; Orellana et al. 2014; Udhya et al. 2014; Nelson et al. 2015). The use of visual pedagogy through visual activity schedules is also a common and efficient practice in children with ASD (Knight et al. 2015), even in the dental domain (Bäckman and Pilebro 1999; Pilebro and Bäckman 2005; Cagetti et al. 2015; Marion et al. 2016). Furthermore, numeric devices, such as the iPad®, are increasingly integrated in the education of children with ASD, and this device has shown some promising results (Kagohara et al. 2013).

Therefore, the aim of this study is to present a training programme for teaching children and adolescents with ASD to be compliant with a dental examination using an iPad® and digital application called çATED that is based on achievement and anxiety.

Materials and methods

Participants

The participants with ASD were recruited in different education centres located in the region of Nantes, France. Information meetings with teams and staff members allowed for presentation of the project, the iPad application, and establishment of the session organisation. Then, a meeting with families was proposed to also present the project, distribute an information letter, and collect signed consent forms from parents. Fifty-five families agreed to participate in the project, and their children were included in the study. Fifty-two children and teenagers with ASD, aged 3–19 years, completed the entire programme over 8 months. Three children were excluded during the programme because 1 child moved from the care centre and 2 children missed more than 25% of the training sessions.

Training program

A visual activity schedule for dental examination, including 6 photos or pictograms, was created. This schedule sub-divided the dental exam into the following 6 simple, progressive steps: 1: sitting on a chair, 2: light shining on the mouth, 3: opening the mouth, 4: placing a mirror in the mouth, 5: placing an explorer on the teeth, and 6: placing both the mirror and explorer in the mouth (Fig. 1).

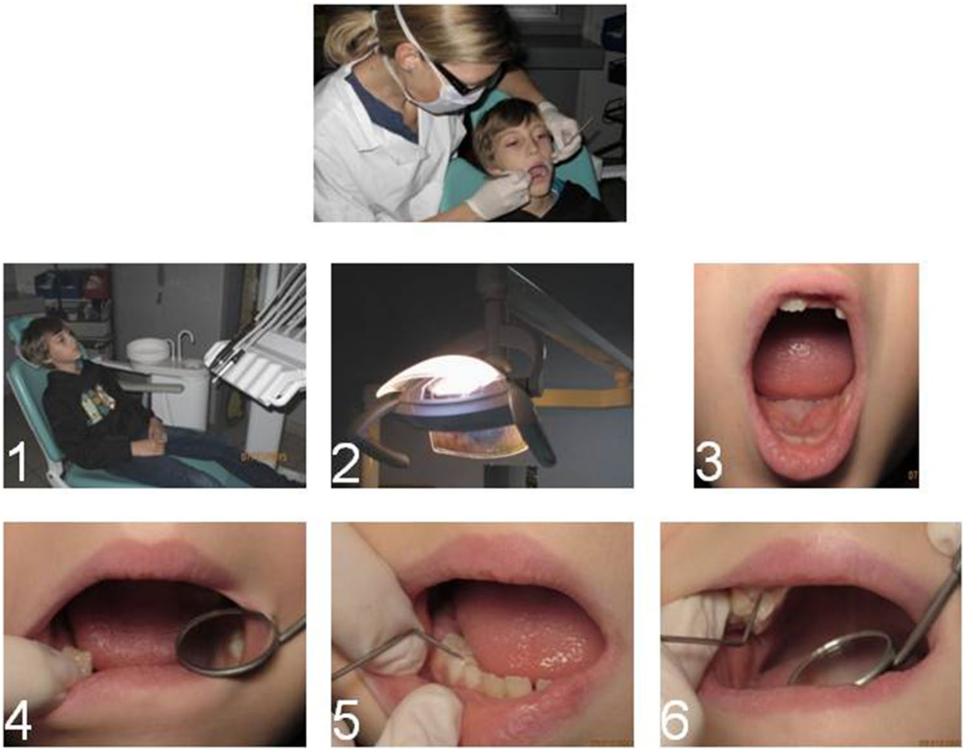

Fig. 1 Example of the visual activity schedule for dental examination at T0 (baseline). 1: sitting on a chair, 2: light shining on the mouth, 3: opening the mouth, 4: placing a mirror in the mouth, 5: placing an explorer on the teeth, and 6: placing both the mirror and explorer in the mouth

The visual activity schedule for the dental exam was integrated on an iPad® using an application called çATED. The çATED app is a digital diary in which pictograms or photos and schedules can be personalised. It was created to help people with autism organise their activities, learn new skills and be more independent. We used the function of decomposition of a complex task into several easier steps for completion. The research team developed several pictograms and photos. The most relevant were chosen with and by children and/or caregivers and then integrated into the application on iPad®. The training programme was conducted at school or in the care centre. During 8 months, dental exams were conducted with the patient sitting on a chair once a month.

Evaluation

The participants were evaluated by a dentist at the beginning of the study (baseline, T0) and every two months (T1, T2, T3, and T4) for 8 months. Evaluations occurred at the school or care centre and used a set of grids and scales to score each step of the dental examination to assess the programme efficacy.

The 6 pictograms use 6 steps based on the following scores that assess the achievement of the dental exam: 1: the step was not completed (the step was not acquired); 2: the step was completed, the child was not fully compliant (the step was currently being acquired); and 3: the step was completed easily (the step was acquired).

Anxiety was also evaluated during the 6 steps of oral exam using the Frankl scale with the highest score indicating the lowest anxiety as follows: 1: definitively negative, 2: negative, 3: positive, and 4: definitively positive.

The score of the 6 steps can be independently studied and grouped. A global score was calculated by adding scores obtained in each step and then dividing by the numbers of steps, i.e., 6. These scores were called SAch (score achievement representing the global achievement of dental exam) and SAnx (score anxiety representing the global anxiety during the exam).

Statistical analysis

Mean scores were calculated for each variable for *achievement* and *anxiety*. Due to the nature of the design and data, which were not normally distributed, the paired sample Wilcoxon signed rank test was used to compare the results and determine the significance of differences between variables. The Bravais Pearson test was used to study the correlation between variables (Dancey and Reidy 2007). Statistical software SPSS version 16.0 was used for statistical analysis.

Results

The study sample included 7 girls and 45 boys, with a mean age of 10.2 years; 19% of the children were younger than 6 years of age, 39% were 6–12 years old, and 42% were 12–19 years old.

Baseline

Dental examination is considered acquired by the child for a score of 3 in all 6 evaluated steps. At the beginning of the study (T0), based on evaluation, 25% of the study sample (13 individuals) had acquired the achievement of a dental exam. Opening the mouth and allowing the use of instruments in the mouth was less successful in achievement than in the first two steps (Fig. 2a). The use of the explorer seemed to be more difficult.

The same pattern was observed for anxiety, the last four steps seemed to be more anxiogenic and the explorer was more anxiogenic than the mirror (based on the score) (Fig. 2b). The dental examination was not considered anxiogenic for the child who scored 4 in all the 6 evaluated steps. Only 7.7% of the sample (4 individuals) was not anxious (score of 4 at each step) during the dental examination at T0.

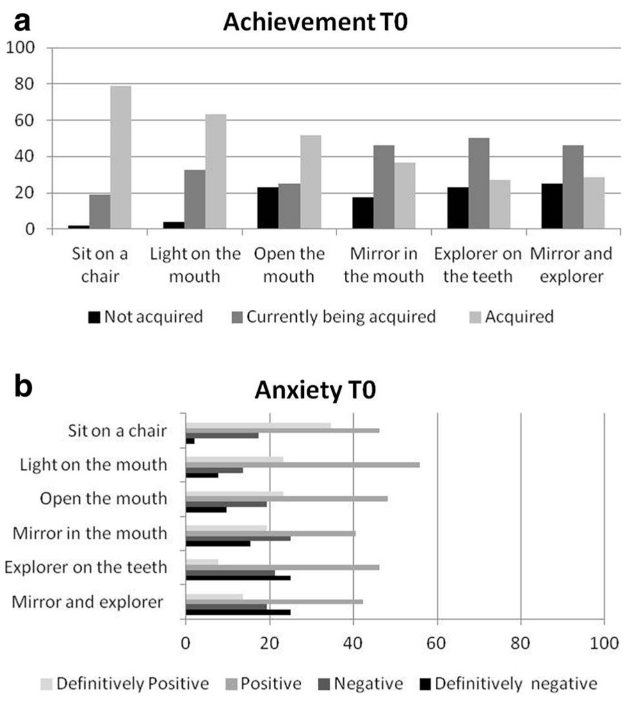

Fig. 2 Initial situation at T0 (at baseline). The 6 steps of the dental exam are described with different scores for achievement (**a**) and anxiety (**b**), as detailed in the methods

Longitudinal survey

The present study results showed that the sample improved their tolerance to the dental examination. During several evaluations, more acquired steps were observed, fewer steps that were not performed and less anxiety (Figs. 3, 4).

The percentage of children not performing the first four steps varied from 2 to 17% at the beginning of the study,

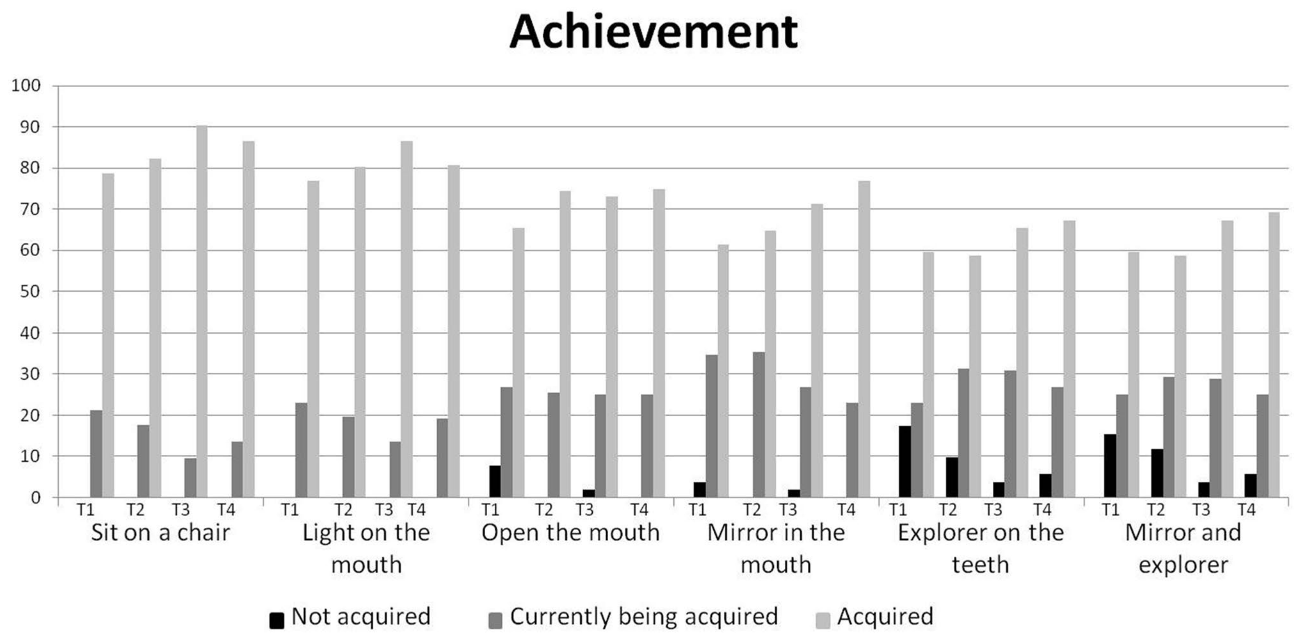

Fig. 3 Longitudinal survey of the achievement of the dental examination. The 6 steps of the dental exam are described with the different scores detailed in methods for achievement. T1: 2 months after the beginning of the experiment, T2: 4 months after the beginning of the experiment, T3: 6 months after the beginning of the experiment, and T4: 8 months after the beginning of the experiment

Fig. 4 Longitudinal survey of the anxiety during the dental examination. The 6 steps of the dental exam are described with different scores detailed in the methods for anxiety. T1: 2 months after the beginning of the experiment, T2: 4 months after the beginning of the experiment, T3: 6 months after the beginning of the experiment, and T4: 8 months after the beginning of the experiment

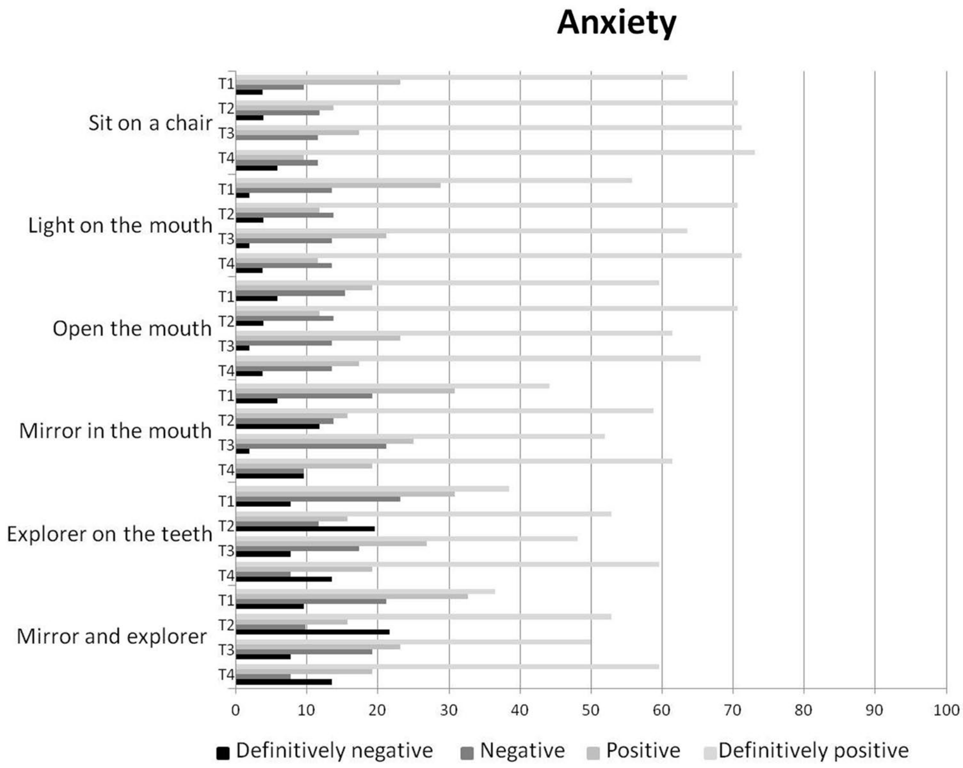

while it decreased down to 0 by the end. Progression was also observed if the percentage of individuals who acquired the 4 steps were considered; they were between 37 and 79% at T0 and between 75 and 86.5% at T4. For the last two steps, evolutions followed the same pattern, which were 27 and 29% success at the beginning and 67 and 69% success at the end of the programme (Fig. 3). However, at T4, 5.8% had not acquired the two steps.

The percentage of individuals who had acquired the entire exam procedure increased over time; it was 25% at the beginning of the study and 65.4% after 8 months.

Regarding anxiety, the same pattern was also observed over time. The percentage of individuals with scores of 1 and 2 (definitively negative and negative) decreased for each step, and those with scores of 3 and 4 increased. Therefore, anxiety seemed to decrease over time (Fig. 4). Furthermore, the percentage of participants who were not anxious (score of 4 in all steps) about the dental examination increased for later steps with values of 36.5% at T1, 51.9% at T2 and T3, and 59.6% at T4.

Statistical analysis

The study results showed an increase in all variable means (step means and SAch and SAnx means) (Table 1). The sample had increased achievement of the exam and was less anxious over time. However, the means of the last steps of the dental exam were lower compared to the means of the first steps.

The positive evolution was statistically significant, as shown by the results of the Wilcoxon test. Over the course of the study, the dental exam was better performed and less anxiety was observed while performing this activity. This significant increase in achievement was seen between T0 and T1, between T2 and T3 and between T0 and T4 for SAch. Regarding SAnx, the difference was significant between T0 and T1 and between T0 and T4 (Table 2). If each step between T0 and T4 is considered, the difference was always significant (Table 3). The study sample progressed in all steps for achievement, and the individuals became less anxious.

Statistical analysis also demonstrated that all steps of the dental exam were correlated, as shown by the Bravais Pearson test results. Globally, the first two steps were more easily performed and were less anxiogenic than the intrusion in the mouth at T0 and T4. The use of the explorer was significantly more difficult and more anxiogenic (Table 4).

Table 1 Means and standard deviations (SDs) of the different dental exam variables at the different evaluation times

Means (standard deviations)	Scores of achievement					Scores of anxiety				
	T0	T1	T2	T3	T4	T0	T1	T2	T3	T4
Sit on a chair	2.8 (0.5)	2.8 (0.4)	2.8 (0.4)	2.9 (0.3)	2.8 (0.4)	3.1 (0.8)	3.5 (0.8)	3.5 (0.9)	3.7 (0.8)	3.5 (0.9)
Light on mouth	2.6 (0.6)	2.8 (0.4)	2.8 (0.4)	2.8 (0.3)	2.8 (0.4)	2.9 (0.8)	3.4 (0.8)	3.5 (0.9)	3.5 (0.8)	3.5 (0.9)
Open mouth	2.3 (0.8)	2.6 (0.6)	2.7 (0.4)	2.7 (0.5)	2.7 (0.4)	2.8 (0.9)	3.3 (0.9)	3.5 (0.9)	3.4 (0.8)	3.4 (0.9)
Mirror in mouth	2.2 (0.7)	2.6 (0.6)	2.6 (0.5)	2.7 (0.5)	2.8 (0.4)	2.6 (1)	3.1 (0.9)	3.2 (1.1)	3.3 (0.9)	3.3 (1)
Explorer on teeth	2 (0.7)	2.4 (0.8)	2.5 (0.7)	2.6 (0.6)	2.6 (0.6)	2.4 (1)	3 (1)	3 (1.2)	3.2 (1)	3.3 (1)
Mirror and explorer	2 (0.7)	2.4 (0.8)	2.5 (0.7)	2.6 (0.6)	2.6 (0.6)	2.4 (1)	3 (1)	3 (1.2)	3.2 (1)	3.3 (1)

T0 at baseline, *T1* at 2 months, *T2* at 4 months, *T3* at 6 months, *T4* at 8 months

Table 2 Means and standard deviations (SDs) of the global score created for achievement (SAch) and anxiety (SAnx) and Wilcoxon test analysis between the different times of evaluation

	SAch					SAnx				
	T0	T1	T2	T3	T4	T0	T1	T2	T3	T4
Mean (SD)	2.3 (0.6)	2.5 (0.5)	2.7 (0.5)	2.7 (0.4)	2.8 (0.6)	2.7 (0.8)	3.2 (0.9)	3.3 (1)	3.4 (0.8)	3.4 (0.9)
Wilcoxon test	T0–T1	T1–T2	T2–T3	T3–T4	T0–T4	T0–T1	T1–T2	T2–T3	T3–T4	T0–T4
Z p	− 4.19***	− 1.3 NS	− 2.15*	− 2.13 NS	− 5.10***	− 4.93***	− 0.89 NS	− 0.90 NS	− 0.58 NS	5.02***

T0 at baseline, *T1* at 2 months, *T2* at 4 months, *T3* at 6 months, *T4* at 8 months, *SAch* score for achievement, *SAnx* score for anxiety

*$p < 0.05$; **$p < 0.01$; ***$p < 0.001$; *NS* non-significant = $p > 0.05$

Table 3 Wilcoxon test analysis for each step of the dental exam between the beginning (T0) and end (T4) of the training programme

Wilcoxon analysis	Achievement		Anxiety	
	Z	p	Z	p
Sit T0 versus Sit T4	– 1	NS	– 2.64	**
Light on mouth T0 versus light on mouth T4	– 2.36	*	– 3.99	***
Open mouth T0 versus open mouth T4	– 4.23	***	– 4.49	***
Mirror in mouth T0 versus mirror in mouth T4	– 4.75	***	– 4.62	***
Explorer on teeth T0 versus explorer on teeth T4	– 4.91	***	– 5.04	***
Mirror and explorer T0 versus mirror and explorer T4	– 4.94	***	– 4.94	***

T0 at baseline, *T4* at 8 months

*$p<0.05$; **$p<0.01$; ***$p<0.001$; *NS* = non-significant = $p>0.05$

Table 4 Wilcoxon test and Bravais Pearson test analysis of achievement and anxiety between the different steps of the dental exam at the beginning (T0) and end (T4) of the training programme

Wilcoxon and Bravais Pearson analysis	Achievement				Anxiety			
	T0		T4		T0		T4	
	Z	r	Z	r	Z	r	Z	r
Sit versus light on the mouth	– 3.8***	0.43**	– 2.45*	0.70***	– 2.87**	0.69***	– 1.34 NS	0.94***
Sit versus open the mouth	– 2.32*	0.52***	– 1.73 NS	0.82***	– 2.67**	0.82***	0 NS	0.98***
Sit versus mirror in the mouth	– 4.67***	0.43**	– 3.81**	0.74***	– 4.01***	0.62***	– 2.5*	0.88***
Sit versus explorer on the teeth	– 5.04***	0.31*	– 3.21***	0.62***	– 5.03***	0.58***	– 2.97**	0.84***
Sit versus mirror and explorer	– 4.92***	0.31*	– 3.05**	0.64***	– 4.65***	0.60***	– 2.97**	0.84***
Light on the mouth versus open the mouth	– 0.42 NS	0.59***	– 1.73 NS	0.85***	– 1.4NS	0.84***	– 1.73 NS	0.96***
Light on the mouth versus mirror in the mouth	– 4***	0.58***	– 1.41 NS	0.90***	– 3.36***	0.80***	– 2.71**	0.90***
Light on the mouth versus explorer on the teeth	– 4.47***	0.43**	– 2.71**	0.70***	– 4.52***	0.70**	– 2.97**	0.84**
Light on the mouth versus Mirror and explorer	– 4.47***	0.46***	– 2.53*	0.72***	– 4.1***	0.73***	– 2.97**	0.84***
Open the mouth versus mirror in the mouth	– 1.3 NS	0.77***	– 0.58 NS	0.85***	– 3.05**	0.88***	– 2.12*	0.93***
Open the mouth versus explorer on the teeth	– 2.98***	0.75***	– 2.12**	0.78***	– 4.46***	0.78***	– 2.64**	0.90***
Open the mouth versus mirror and explorer	– 2.84**	0.72***	– 2.45*	0.73***	– 4***	0.81***	– 2.64**	0.90***
Mirror in the mouth versus explorer on the teeth	– 2.31*	0.79***	– 2.64**	0.82***	– 3.5***	0.87***	– 1.63 NS	0.95***
Mirror in the mouth versus mirror and explorer	– 2.33*	0.80***	– 2.45*	0.85***	– 2.89**	0.90***	– 1.63 NS	0.95***
Explorer on the teeth versus mirror and explorer	0 NS	0.96***	– 5.06***	0.97***	1.63 NS	0.95***	0 NS	1***

*$p<0.05$, **$p<0.01$, ***$p<0.001$, and *NS* non-significant with $p>0.05$

Discussion

Autism spectrum disorder is a complex, pervasive, heterogeneous condition with multiple sub-types and developmental trajectories that lead often to poor oral health. The main goal of the "çATED pour tes dents" project was to improve oral health in children and teenagers with ASD. The training programme was implemented in school or at a care centre. For 8 months, each participant met with the research team once per week to learn about tooth brushing, and a dental exam was conducted on a chair once per month. The other weekdays, caregivers used the app to oversee tooth brushing after lunch, which helped familiarise individuals with the device. In this report, a part of the project, the in vivo desensitisation of dental examination, is presented.

Some authors have previously demonstrated interest in a training programme for dental care in individuals with ASD. In 1996, Luscre and Center (1996) used a combination of desensitisation, video modelling and reinforcement to reduce fears about dental care. Cagetti et al. (2015) used visual support for different dental care protocols to help children with ASD undergo oral examination and treatments. The originality of the present training programme consists of the association of two efficient strategies, a behavioural approach and visual pedagogy, with a promising tool as a mediator, a digital tablet. An iPad® was used with the hypothesis that it could be a mediator between children with ASD and the dentist because it has multiple advantages. First, the iPad® is easy to use and is a common and familiar tool for typically developing children and adolescents as well as for those with ASD. Second, the iPad® is also an attractive device

for children with ASD and could be considered as positive reinforcement. The device was chosen with the hypothesis that it would facilitate children's motivation and social communication (Karsenti and Fievez 2013; Heitz 2015). The iPad® contributes to behavioural support. This was also demonstrated for the digital diary çATED that was used in this present study; Mercier et al. (2016) showed that this app enhanced communication between children with ASD and between children with ASD and adults. Third, the iPad® can support visual pedagogy. Marion et al. (2016) showed that parents often prefer a numeric device to a traditional (paper) one. The çATED app allowed for the creation of visual activity schedules that are evidence-based practices for people with ASD to learn skills (Koyama and Wang 2011; Knight et al. 2015).

Data from 52 children and adolescents were analysed in this study. The sample could be considered as a convenience sample because it was elaborated without a probability sampling technique, i.e., individuals were not randomly selected. However, the following inclusion criteria were used: clinical diagnosis of ASD, participating in one of the educational locations in the study, and written consent from their parents. Moreover, the study sample included 7 girls and 45 boys, which is representative of the gender ratio of ASD. The sample is also representative of ASD because it included children with mild to severe autism.

Regarding dental examination, the study population increased over time. The following indicators supported this improvement: (1) the percentage of children acquiring one step increased over time, (2) the percentage of children acquiring the entire dental examination process increased during the experiment, (3) the mean of achievement scores for each stage significantly increased, and (4) the mean global achievement scores significantly increased over time.

Regarding anxiety, a similar evolution was globally observed. The Frankl scale was used, which has been the most frequently used in the dental field to evaluate anxiety and compliance in ASD patients. The majority of the study population was not relaxed at the beginning of the experiment. This was previously shown by other authors (Barry et al. 2014; Orellana et al. 2014). Orellana et al. (2014) reported that 73.5% of children with ASD showed a reluctant behaviour during the pre-test. Over time, anxiety decreased in the present study, which agrees with the findings of Orellana et al. (2014).

In the present study, failure and anxiety decreased, indicating that the training programme was effective and agreed with prior reports that training programmes are useful and promote better compliance with dental sessions (Cuvo et al. 2010; Orellana et al. 2014; Cagetti et al. 2015). Progress was visible at the first re-evaluation at month 2; however, the population's progress was not linear. The latter might be linked to a stabilisation stage of assets. The population performed better, while maintaining its assets; for instance, children acquired steps and when a step was acquired at an evaluation (opening the mouth, for example), the child generally maintained this step at the next evaluation. The program's length (8-month training) was likely to be sufficient to maintain those achievements and increase the number of children progressing.

Even if examination steps were linked to one another and were considered as a whole, differences were observed between steps. The percentages varied depending on specific stages. However, some of the examination steps (introduction of instruments in the mouth) were less successful and generated more anxiety throughout the programme, even if these differences faded over time. When evaluating the achievement and anxiety of dental exam at time T, we observed a global decrease in achievement scores, and differences were often statistically significant. Two hypotheses can be raised to explain this result.

The first hypothesis involves the fatigability of the study population. This could be in accordance with the previous study by Ash, which highlights that the attention and concentration of children decrease during a session. Often, children succeed less in the end stages of the experiment than in the beginning (Ash 1946).

The second hypothesis is that the increased difficulty is linked to procedures where tools are placed in the children's mouth. There were challenges in mouth opening, and introducing instruments produced more failures in achievement and more anxiety, even if there was more success at the end of the programme than at the beginning. This avoidance/escape from aversive dental stimuli was previously described in the literature, including by Cuvo et al. (2010).

The stage of "sitting on a chair" was not difficult for the study population, and failures at this stage were rare. However, generalising this step in a dental office could have somewhat different results. Indeed, this research was conducted within educational centres, in places that were familiar for the children, and changing the locale could affect the results. Furthermore, children in the present study had to sit down on a chair, while the dentist's chair was more impressive and reclines, which could also influence the results.

The stage "light shining on the mouth" posed a few challenges. Children with ASD often have sensory disorders that make this stage challenging. In the literature, many studies have highlighted the sensory profile of people with ASD and have noted the fact that the dental environment (light, sound, and smell) could create sensory discomfort that requires adaptation (Stein et al. 2011; Cermak et al. 2015). In the present study, the light (headlamp or mobile phone) did not produce the same power and intensity as a dental office light, which could explain the results.

The stage "opening the mouth" had significant differences at the beginning of the programme from the two previous

steps as well as with the "sitting on a chair" step towards the end of the programme. Intrusion in the child's personal space started at this stage, which might explain the difficulties in achievement. This hypothesis also explains the lower success rate in the last three stages of the dental examination.

"Having an instrument in the mouth" was harder to perform for the sample and using a sharp and noisy instrument (explorer) made it even more difficult at the beginning, which improved over time. The challenge was even stronger when introducing two instruments, which may be due to greater obstruction linked to the use of two instruments as well as to anxiety about the presence of the explorer.

Overall, dental exam progress was observed. The programme presented here was effective; however, individuals progressed at different paces, and some did not acquire achievement of the dental exam after a time period of 8 months and/or were still anxious. For these individuals, a more intensive programme could have been more effective. Bishop et al. (2013) used a 30-step stimulus fading hierarchy to increase compliance with tooth brushing. The same technique could be used with the mirror and explorer to familiarise patients with these instruments and to easily improve their introduction into the mouth without generating anxiety. This could be a good approach for enhancing compliance in the most reluctant children as well as allow for faster progress.

Conclusions

Training children and adolescents with ASD to comply with an oral assessment is necessary. Using the çATED application in a dental context within a training programme improved the oral health of the study sample by contributing to in vivo desensitisation of the dental exam. The iPad® is an easy and attractive device; today, it is also routinely used by people with ASD. This tool supports the development of new strategies for professionals (dentists, caregivers, etc.) and for children and teenagers with ASD. The approach presented here is efficient for helping children with ASD learn a new skill, even in a dental context.

Acknowledgements This study was supported by grants from the International Foundation of Applied Disability Research (FIRAH). We would like to express our gratitude to the çATED-autisme team. The authors would like to thank children, parents and professionals who participated to the "çATED pour tes dents" program.

Compliance with ethical standards

Ethical standards All procedures performed in this study involving human participants were in accordance with the ethical standards of the institutional and/or national research committee and with the 1964 Helsinki Declaration and its later amendments or comparable ethical standards.

Informed consent For this study, written informed consent was obtained from all parents of children and adolescents included in the study.

Conflict of interest The authors declare that they have no conflicts of interest.

References

American Academy of Pediatric Dentistry. Guideline on behavior guidance for the pediatric dental patient. Pediatr Dent. 2016;38 (6):185–98.

American Psychiatric Association. Diagnostic and statistical manual of mental disorders. 5th ed. Washington DC: American Psychiatric; 2013.

Ash ES. Forming impressions of personality. J Abnorm Soc Psychol. 1946;41:258–90.

Bäckman B, Pilebro C. Visual pedagogy in dentistry for children with autism. ASDC J Dent Child. 1999;66 (5):325–31.

Barry S, O'Sullivan EA, Toumba KJ. Barriers to dental care for children with autism spectrum disorder. Eur Arch Paediatr Dent. 2014;15:127–34.

Bishop MR, Kenzer AL, Coffman CM, et al. Using stimulus fading without escape extinction to increase compliance with toothbrushing in children with autism. Res Autism Spec Disord. 2013;7:680–6.

Brickhouse TH, Farrington FH, Best AM, Ellsworth CW. Barriers to dental care for children in Virginia with autism spectrum disorders. J Dent Child (Chic). 2009;76:188–93.

Cagetti MG, Mastroberardino S, Campus S, et al. Dental care protocol based on visual supports for children with autism spectrum disorders. Med Oral Patol Cir Bucal. 2015;20 (5):e598–604.

Cermak SA, Stein Duker LI, Williams ME, et al. Sensory adapted dental environments to enhance oral care for children with autism spectrum disorders: a randomized controlled pilot study. J Autism Dev Disord. 2015;45 (9):2876–88.

Conyers C, Miltenberger RG, Peterson B, et al. An evaluation of in vivo desensitization and video modeling to increase compliance with dental procedures in persons with mental retardation. J Appl Behav Anal. 2004;37 (2):233–8.

Cuvo AJ, Godard A, Huckfeldt R, Demattei R. Training children with autism spectrum disorders to be compliant with an oral assessment. Res Autism Spect Disord. 2010;4 (4):681–96.

Dancey C, Reidy J. Statistics without math for psychologists. Paris: De Boeck; 2007 (**French**).

Delli K, Reichart PA, Bornstein MM, Livas C. Management of children with autism spectrum disorder in the dental setting: concerns, behavioural approaches and recommendations. Med Oral Patol Oral Cir Bucal. 2013;18:e862.

Gandhi RP, Klein U. Autism spectrum disorders: an update on oral health management. J Evid Based Dent Pract. 2014;14:115–26.

Heitz MH. Clis'Tab: first results of an innovative project. La nouvelle revue de l'adaptation et de la scolarisation. 2015;69 (1):191–206 (**French**).

Hernandez P, Ikkanda Z. Applied behavior analysis: behavior management of children with autism spectrum disorders in dental environments. J Am Dent Assoc. 2011;142:281–7.

Jaber MA. Dental caries experience, oral health status and treatment needs of dental patients with autism. J Applied Oral Sci. 2011;19:212–7.

Kagohara DM, van der Meer L, Ramdoss, et al. Using iPods® and iPads® in teaching programs for individuals with

developmental disabilities: a systematic review. Res Dev Disabil. 2013;34:147–56.

Karsenti T, Fievez AN. The iPad in education: uses, benefits, and challenges—a survey of 6,057 students and 302 teachers in Quebec, Canada. Montreal, QC: CRIFPE; 2013. p. 56 (**report no: 978-2-923808-34-5$4**).

Kemp F. Alternatives: a review of non-pharmacologic approaches to increasing the cooperation of patients with special needs to inherently unpleasant dental procedures. Behav Anal Today. 2005;6:88.

Knight V, Sartini E, Spriggs AD. Evaluating visual activity schedules as evidence-based practice for individuals with autism spectrum disorders. J Autism Dev Disordr. 2015;45:157–78.

Koyama T, Wang HT. Use of activity schedule to promote independent performance of individuals with autism and other intellectual disabilities: a review. Res Dev Disabil. 2011;32:2235–42.

Lai B, Milano M, Roberts MW, Hooper SR. Unmet dental needs and barriers to dental care among children with autism spectrum disorders. J Autism Dev Disord. 2012;42:1294–303.

Lewis C, Vigo L, Novak L, Klein EJ. Listening to parents: a qualitative look at the dental and oral care experiences of children with autism spectrum disorder. Pediatr Dent. 2015;37 (7):98E–104E.

Loo CY, Graham RM, Hughes CV. Behaviour guidance in dental treatment of patients with autism spectrum disorder. Int J Paediatr Dent. 2009;19:390–8.

Luscre DM, Center DB. Procedures for reducing dental fear in children with autism. J Autism Dev Disord. 1996;26 (5):547–56.

Marion IW, Nelson TM, Sheller B, et al. Dental stories for children with autism. Spec Care Dent. 2016;36 (4):181–6.

Marshall J, Sheller B, Mancl L. Caries-risk assessment and caries status of children with autism. Pediatr Dent. 2010;32:69–75.

Mercier C, Bourdon P, Bourdet JF. The time of the child with autism and the time of the professional: adopt the rhythm of the learner in order to facilitate the access to new learning. Distances et médiations des savoirs Distance Med Knowl. 2016;16 (**French**).

Nelson TM, Sheller B, Friedman CS, et al. Educational and therapeutic behavioral approaches to providing dental care for patients with autism spectrum disorder. Spec Care Dent. 2015;35:105–13.

Orellana LM, Martinez-Sanchis S, Silvestre FJ. Training adults and children with an autism spectrum disorder to be compliant with a clinical dental assessment using a TEACCH-based approach. J Autism Dev Disord. 2014;44 (4):776–85.

Pilebro C, Bäckman B. Teaching oral hygiene to children with autism. Int J Paediatr Dent. 2005;15:1–9.

Reichow B, Barton EE, Boyd BA, Hume K. Early intensive behavioral intervention (EIBI) for young children with autism spectrum disorders (ASD). Cochrane Database Syst Rev. 2012;10:CD009260.

Stein LI, Polido JC, Mailloux Z, et al. Oral care and sensory sensitivities in children with autism spectrum disorders. Spec Care Dent. 2011;31 (3):102–10.

Stein LI, Polido JC, Najera SOL, Cermak SA. Oral care experiences and challenges in children with autism spectrum disorders. Pediatr Dent. 2012;34:387–91.

Stein LI, Polido JC, Cermak SA. Oral care and sensory over-responsivity in children with autism spectrum disorders. Pediatr Dent. 2013;35:230–5.

Udhya J, Varadharaja MM, Parthiban J. Autism disorder (AD): an updated review for paediatric dentists. J Clin Diagn Res. 2014;8 (2):275–9.

Weil TN, Inglehart MR. Three-to 21-year-old patients with autism spectrum disorders: parents' perceptions of severity of symptoms, oral health and oral health-related behavior. Pediatr Dent. 2012;34:473–9.
